# Major environmental drivers determining life and death of cold-water corals through time

**DOI:** 10.1371/journal.pbio.3001628

**Published:** 2022-05-19

**Authors:** Rodrigo da Costa Portilho-Ramos, Jürgen Titschack, Claudia Wienberg, Michael Georg Siccha Rojas, Yusuke Yokoyama, Dierk Hebbeln

**Affiliations:** 1 MARUM–Center for Marine Environmental Sciences, University of Bremen, Bremen, Germany; 2 Senckenberg am Meer, Marine Research Department, Wilhelmshaven, Germany; 3 Analytical Center for Environmental Science–Atmosphere and Ocean Research Institute, University of Tokyo, Tokyo, Japan; University of Queensland, AUSTRALIA

## Abstract

Cold-water corals (CWCs) are the engineers of complex ecosystems forming unique biodiversity hotspots in the deep sea. They are expected to suffer dramatically from future environmental changes in the oceans such as ocean warming, food depletion, deoxygenation, and acidification. However, over the last decades of intense deep-sea research, no extinction event of a CWC ecosystem is documented, leaving quite some uncertainty on their sensitivity to these environmental parameters. Paleoceanographic reconstructions offer the opportunity to align the on- and offsets of CWC proliferation to environmental parameters. Here, we present the synthesis of 6 case studies from the North Atlantic Ocean and the Mediterranean Sea, revealing that food supply controlled by export production and turbulent hydrodynamics at the seabed exerted the strongest impact on coral vitality during the past 20,000 years, whereas locally low oxygen concentrations in the bottom water can act as an additional relevant stressor. The fate of CWCs in a changing ocean will largely depend on how these oceanographic processes will be modulated. Future ocean deoxygenation may be compensated regionally where the food delivery and food quality are optimal.

## Abstract 2

Cold-water corals are the engineers of complex ecosystems forming unique biodiversity hot spots in the deep sea, but there is substantial uncertainty regarding their sensitivity to environmental parameters. A synthesis of six paleoceanographic reconstructions reveals that food supply exerted the strongest impact on coral vitality during the past 20,000 years, whereas locally low oxygen concentrations can act as a stressor.

## Introduction

Framework-forming scleractinian cold-water corals (CWCs), such as *Lophelia pertusa* (recently assigned to the genus *Desmophyllum* [1]), play a crucial role in deep-sea biodiversity and ecosystem functioning, regulating the food web structure and nutrient cycling [2,3]. They act as ecosystem engineers, which build complex three-dimensional reef structures that develop over geological timescales to coral mounds encompassing several reef phases [4,5]. The CWC reefs and mounds offer habitat for numerous deep-sea organisms including commercially important fish [6] and, thereby, are important on maintenance of the fish stock and secure (human) food resources from fisheries [7]. CWCs reefs are considered vulnerable marine ecosystems [8]. As they are exposed to escalating anthropogenic threats associated with destructive fishing techniques (e.g., bottom trawling and towed dredges), several marine protected areas (MPAs; e.g., off Norway, Ireland, and around the Azores) have been established to protect CWC ecosystems [9]. Despite the success on reducing mechanical damage of fishing activities [10], MPAs cannot protect CWCs against threats induced by the ongoing climate change [11]. These include ocean warming, acidification, deoxygenation, and decreasing particulate organic matter fluxes to the seabed, which are expected to have a severe impact on marine ecosystems including CWCs [11–16]. Thus, understanding how CWC ecosystems might respond to environmental changes in the future is of pivotal importance to enable knowledge-guided management decisions and mitigation policies to protect these ecosystems and their services for society [11,17].

Global distribution of CWC ecosystems is driven by a combination of different physical–chemical and biological factors. CWCs have been found thriving within a broad range of temperatures (4 to 15°C), salinities (33 to 39 psu), and dissolved oxygen concentrations (0.5 to 6 ml l^−1^) in water depths mainly (but not exclusively) beyond the shelf break [4,5,18–21]. As sessile suspension feeders, CWCs are highly dependent on external food supply, therefore ordinarily developing in areas of elevated primary production [5,22] and/or in areas with energetic bottom-water hydrodynamics [23,24], marked by bottom current velocities of >8 cm s^−1^ [25]. Furthermore, they thrive generally associated with water mass boundaries with a strong density gradient [26], where they take advantage of the accumulation of sinking organic food particles in nepheloid layers as well as of the turbulent lateral flux of suspended food particles promoted by internal (tide) wave propagation [23,25]. In general, CWCs occur in well-ventilated waters with dissolved oxygen concentrations of 4 ml l^−1^ [18], although, recently, small and sparse living colonies as well as flourishing reefs have been found under hypoxic conditions (0.5 to 1.3 ml l^−1^) within the oxygen minimum zones (OMZs) along the western African margin [21,27–29].

Assessing the fate of CWCs under ongoing and future climatic changes is difficult, mostly because of a complete lack of field observations documenting local extinction events of CWCs. Furthermore, the complexity of physiological processes, genotypes and geographic distribution, species-specific ecological preferences and tolerances as well as their time-dependent response to multistressor exposure has been leading to contradictory results in manipulative experiments [19,30–35]. Experimental studies have shown that one parameter can be superimposed on any other, exacerbating or moderating their impact on CWCs as, e.g., increased respiration rates with increased temperatures [19,31], increased growth rates with combined increased temperatures and food supply [30], or insignificant changes in growth rates with simultaneously increased temperatures and CO_2_ concentrations [36]. Currently, no studies are available addressing the effect of salinity changes on CWCs [37]. In this context, a paleoceanographic approach takes advantage of the long-term development of CWC ecosystems and offers the unique opportunity to align frequently reported on- and offsets of CWC proliferation to specific changes in paleoenvironmental conditions [38]. Following such an approach allows for the identification of the key environmental drivers, which were able to push CWC ecosystems beyond a threshold, causing their demise but also their recurrence in the past. Moreover, it offers the opportunity to address the long-term exposure of CWCs to synergistic effects of multiple parameters (multistressor) through time. It enables us to improve our understanding of the response of CWCs to past climate changes and, hence, provides crucial information about their likely response to those changes expected in the future.

**Fig 1:**
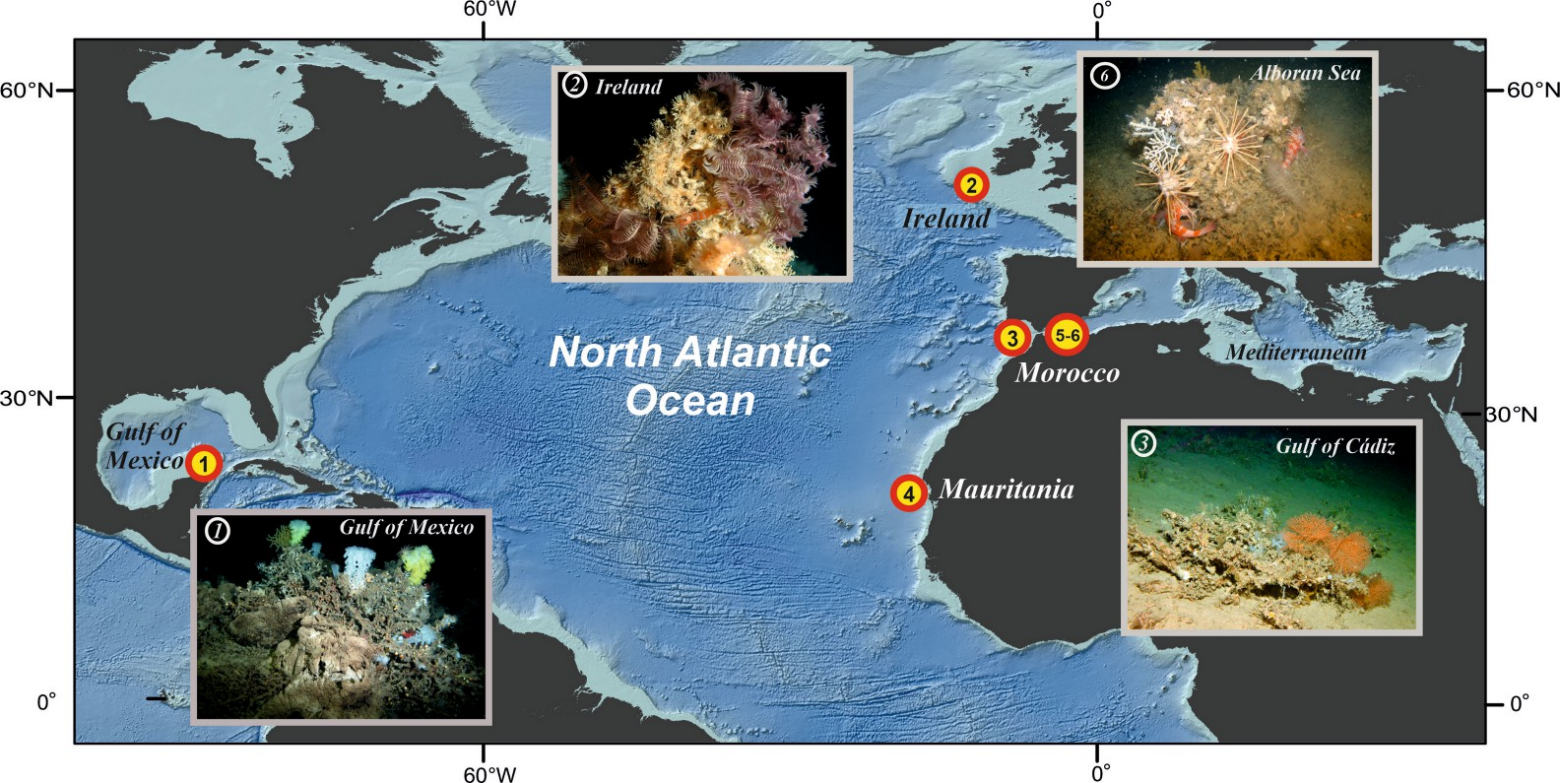
Distribution of case study sites in the North Atlantic Ocean and in the Mediterranean Sea. North Atlantic: (**1**) Gulf of Mexico (Campeche Bank), (**2**) Irish margin (Porcupine Seabight), (**3**) Moroccan margin (Gulf of Cádiz), (**4**) Mauritanian margin; Mediterranean: (**5**) Alboran Sea (West Melilla) and (**6**) Alboran Sea (East Melilla). Map was produced with ESRI ArcGIS 10.7; Source of bathymetry: GEBCO 2019 Grid [59]. *ROV images copyright MARUM ROV Cherokee*, *Bremen*, *Germany*.

In order to identify the most critical physical–chemical parameters having controlled the demise and/or recurrence of CWCs in a given region, we provide the first basin-wide and comprehensive database of paleoenvironmental parameters assumed to have played a paramount role on the spatial–temporal development of CWCs over the past approximately 20,000 years before present (20,000 years = 20 kyr BP). This interval of time comprises the last major global warming event associated with the transition from the last glacial period to the present interglacial [39], which is characterized by a sea level rise of around 120 m [40] and a large-scale reorganization of the oceanic-atmosphere system [41,42]. Here, we reconstructed the bottom-water conditions using conventional paleoceanographic proxies from marine sediments collected from 6 locations in the North Atlantic Ocean and the Mediterranean Sea at water depths between 360 and 900 m (Fig 1). Our study is based on comparing the temporal occurrence of *L*. *pertusa*, the most abundant reef-building CWC species in the Atlantic Ocean, as obtained from sediment cores retrieved from CWC mounds (on-mound cores) with the ambient paleoenvironmental conditions obtained from undisturbed sediment cores from nearby settings (off-mound cores) following the approach outlined by [38]. Previously, such studies have shown that alternating phases of coral presence and absence usually co-occur with regional paleoenvironmental changes (e.g., [43–48]). Based on these studies, here, we apply for the first time a coherent set of paleoceanographic proxies addressing the main suspected controlling parameters for *L*. *pertusa* to the 6 case studies selected (Fig 1).

## Material and methods

### Selection of case studies

To assess the impact of changing paleoenvironmental conditions on the development of CWC ecosystems, we compiled well-documented records from 6 CWC sites in the North Atlantic and in the Mediterranean Sea revealing at least one major change (i.e., on- or offset) in CWC proliferation: (**1**) Gulf of Mexico (Campeche Bank; [49]), (**2**) Irish margin (Porcupine Seabight; [44,45,50]), (**3**) Moroccan margin (Gulf of Cádiz; [45,51,52]), (**4**) Mauritanian margin [53,54], and 2 sites in the Alboran Sea in the western Mediterranean Sea: (**5**) West Melilla [55] and (**6**) East Melilla [56–58]. In order to provide a comprehensive data set for all regions, we combined already published records with newly established data. Details on the sediment cores used and an overview about already existing and newly established data sets are provided in S1 and S2 Tables. In the following, each of the applied methods is briefly introduced; more details are provided in S1 Text.

### The geological record of regional CWC demise and recurrence

Still, it is not fully understood why in a given area CWCs thrive at one place and are absent at a nearby site, although the general environmental setting should be favorable at both sites. However, when such favorable conditions prevail, coral mounds seem to be the most preferred sites for CWC settlement as documented by the repetitive formation phases found for many coral mounds [43,44,54,59,60]. Consequently, coral mound records probably provide the most complete record of past coral occurrence and its response to changing environmental conditions. In this context, the spatial distribution used in ecological studies (presence/absence patterns) is replaced in this geological approach by the temporal distribution at one site, in which periods without coral growth basically represent the “spatial absence” used in ecology.

**Fig 2:**
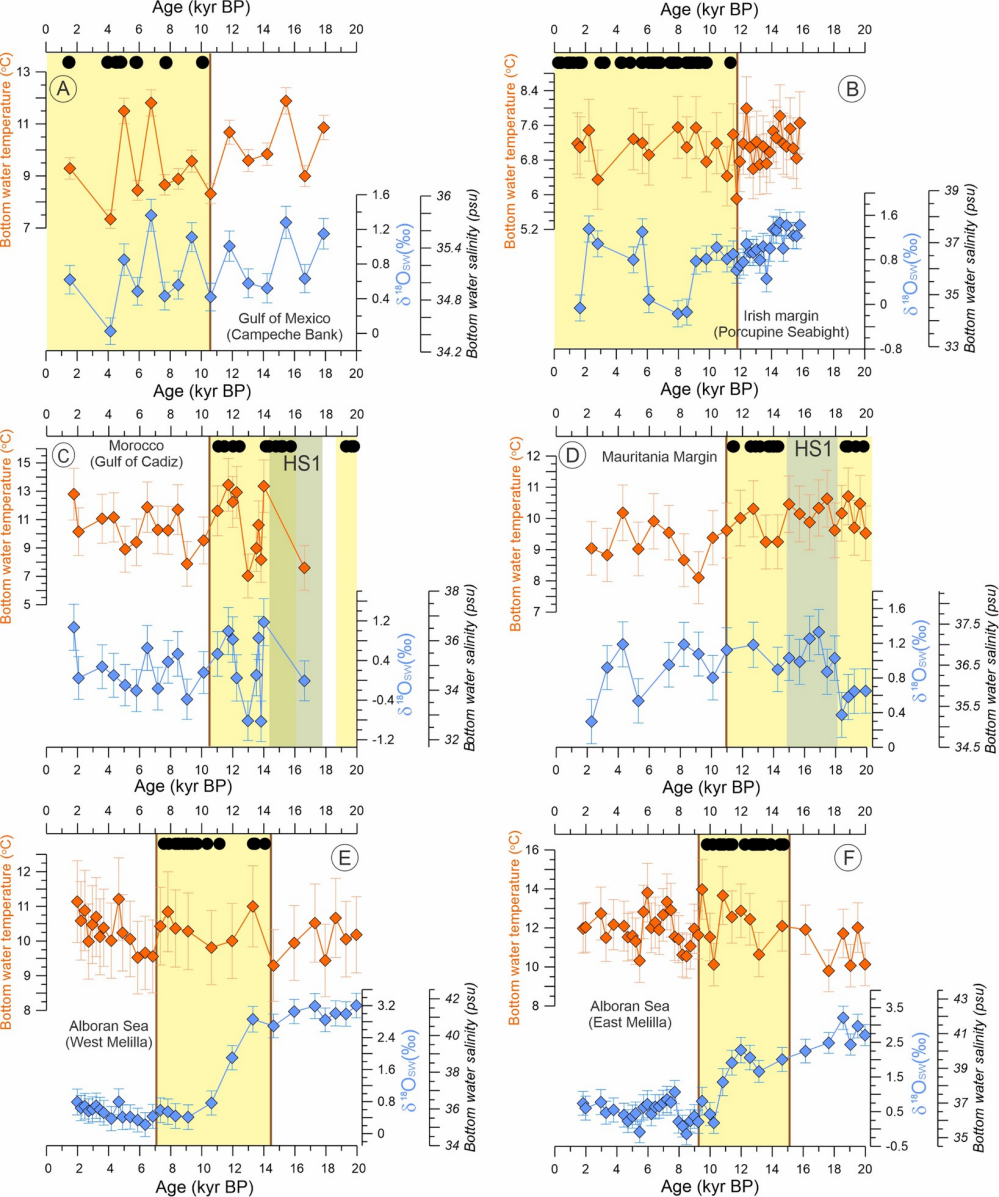
Long-term development of *Lophelia pertusa* in the North Atlantic and the Mediterranean Sea superimposed on the temperature and salinity changes during the last 20 kyr. The occurrence of *L. pertusa* in each region is indicated by black dots at the top, reflecting coral fragments dated by the U/Th and/or AMS^14^C method (see references in the S2 Table), while the yellow vertical bars highlight periods of *L*. *pertusa* proliferation with their limits defining on- and offsets of coral growth (vertical brown lines). Bottom-water temperature and salinity were estimated using paired Mg/Ca ratio and stable oxygen isotope (δ^18^O) measurements on benthic foraminifera shells (see S1 Text). While bottom-water temperature is calculated from Mg/Ca ratios (for details, see S4 Table), the δ^18^O_sw_ record is used here as a proxy for salinity. The greenish bar indicates the period of HS, which (partly) overlaps with periods without coral growth off Mauritania and Morocco. The brown vertical line indicates the transition between on and offset of CWC proliferation. The underlying data for this figure can be found in https://doi.org/10.1594/PANGAEA.932775. BP, before present; HS1, Heinrich Stadial 1.

**Fig 3:**
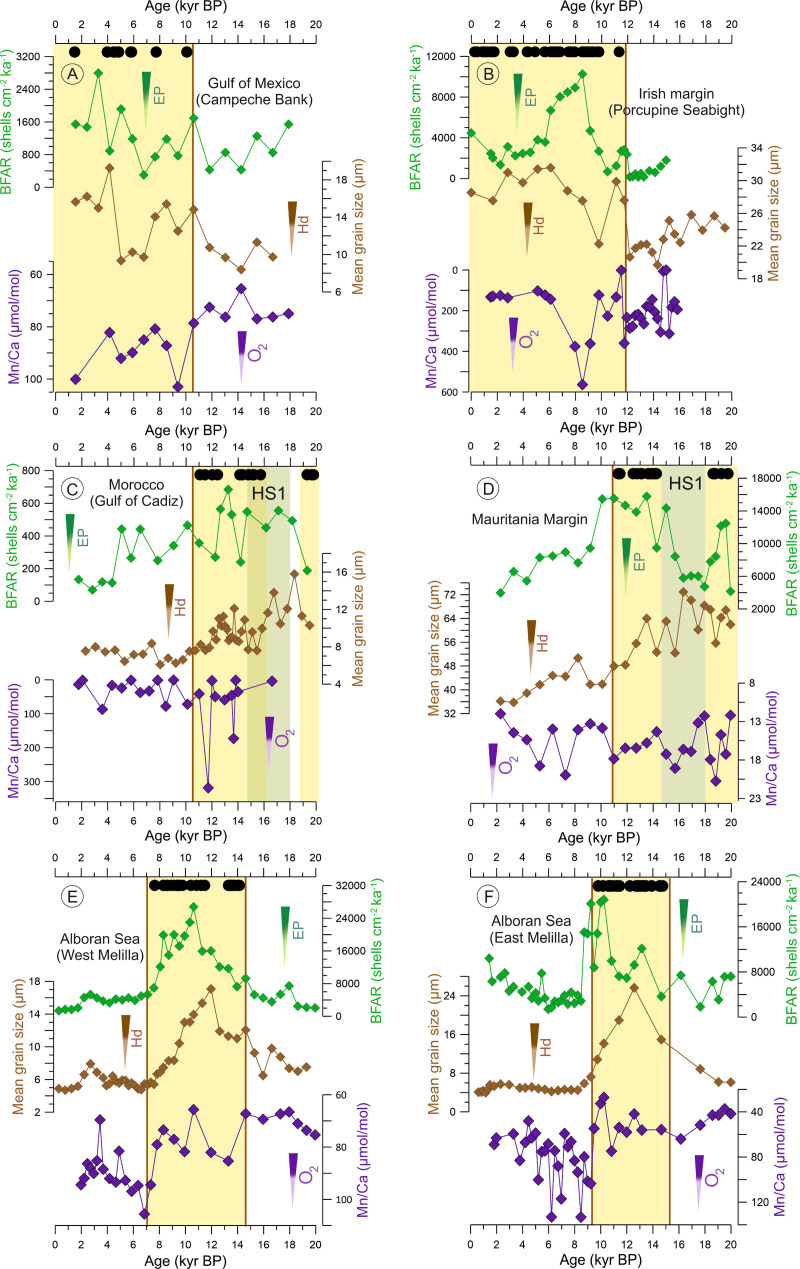
The long-term development of *Lophelia pertusa* in the North Atlantic and the Mediterranean Sea superimposed on food supply, bottom currents proxies, and bottom-water oxygenation during the last 20 kyr. The occurrence of *L. pertusa* in each region is indicated by black dots at the top, reflecting coral fragments dated by the U/Th and/or AMS^14^C method (see references in S2 Table), while the yellow vertical bars highlight periods of *L*. *pertusa* prolific phase with their limits defining on- and offsets of coral growth (vertical brown lines). The BFAR (green) is used to reconstruct the organic matter supply to the seafloor [74], while the mean grain size (brown) is used as a proxy for the bottom-water Hd [44,75]. The bottom-water oxygenation (O_2_) was estimated qualitatively by the benthic foraminifera Mn/Ca ratio (note the inverse axis) following [71]. The greenish bar indicates the period of HS1, which (partly) overlaps with periods without coral growth off Mauritania and Morocco. The brown vertical line indicates the transition between on and offset of CWC proliferation. The underlying data for this figure can be found in https://doi.org/10.1594/PANGAEA.932775. BFAR, benthic foraminifera accumulation rate; BP, before present; EP, export production; Hd, hydrodynamics; HS1, Heinrich Stadial 1; O_2_, Oxygen.

To define periods of coral presence, *L*. *pertusa* fragments have been taken from the on-mound cores and dated either by accelerator mass spectrometry radiocarbon dating (AMS ^14^C) or by U/Th dating. All coral ages have been previously published (see S3 Table). The well-dated coral fragments reveal a regional asynchrony in the temporal development of *L*. *pertusa* in individual areas of the North Atlantic and the Mediterranean Sea (Figs 2 and 3, S7–S12 Figs). In the Gulf of Mexico (Campeche Bank) and along the Irish margin (Porcupine Seabight), the most recent period of prolific *L*. *pertusa* occurrences started at the onset of the Holocene around 10 to 11 kyr BP [45,49,50] (Figs 2 and 3, S7 and S8 Figs). Simultaneously, *L*. *pertusa* disappeared from the Moroccan (Gulf of Cádiz) and Mauritanian margins with the onset of the Holocene, while they have been most prolific during the last glacial period, although a period of coral absence largely overlapping with the millennial-scale paleoclimatic event named as Heinrich Stadial 1 (HS1; 14.7 to 18 kyr BP) was documented (Figs 2 and 3, S9 and S10 Figs) [45,52–54]. *Lophelia pertusa* reoccurred in the Mediterranean Sea (Alboran Sea, West, and East Melilla coral mound provinces) with the onset of the Bølling–Allerød interstadial at around 14 kyr BP (Figs 2 and 3, S11 and S12 Figs) and remained most prolific until the Early Holocene. Afterward, it became progressively less abundant [56–58,61] and locally even completely disappeared (West Melilla: at approximately 5 kyr BP [55]).

### Reconstruction of the regional paleoenvironmental conditions

The off-mound cores collected in the 6 study areas (Fig 1) have been investigated by applying well-established paleoceanographic proxies. The focus was on proxies describing the environmental setting in the benthic realm to reconstruct changes affecting the CWC ecosystems. Of all paleoenvironmental parameters considered here, only the grain-size data used as a proxy for the hydrodynamic setting can be directly affected by the distance between the off-mound site and the coral mound site. However, even though coral mounds (being able to accelerate local currents due to their elevated topography, e.g., [62]) and thriving coral reefs on their top (being able to decelerate local currents due to the baffling effect of the coral framework, e.g., [63]) may induce local effects, their ability to modulate the regional current regime is limited. Hence, the regional hydrodynamic setting is best recorded in the off-mound records that are generally unaffected by local coral mound-related effects. The reconstruction of the bottom-water hydrodynamic conditions is complemented by reconstructions of bottom-water temperatures, salinities and oxygen conditions as well as organic matter flux to the seafloor. Hereby, we partially relied on already published data (see S2 Table). It is noteworthy that our approach does not allow to cover the effects of ocean acidification as the last major global warming event considered here does not provide any equivalent change in ocean pH conditions as it is expected until the end of this century [64].

#### Age models

Most of the off-mound cores are presented in their original published chronologies based on linear interpolation of AMS ^14^C ages obtained from mixed planktonic foraminifera (see S2 Table). In addition to published age models, new AMS ^14^C data from the Irish (Porcupine Seabight) and Mauritanian margins are presented here, as these cores so far lacked any age model (for details, see S4 Table). The 7 new raw AMS ^14^C ages were calibrated using the software PaleoDataView version 0.8.3.5 [65], with the Incal20 radiocarbon calibration curve [66] and a variable simulated reservoir age from transient modeling experiments described in Butzin and colleagues [67]. The calibrated ages can be found in S4 Table. Further details on the generation of these age models are provided in S1 Text.

#### Bottom-water temperatures, salinities, and oxygen conditions based on element/Ca ratios (Mg/Ca and Mn/Ca) and stable oxygen isotopes (δ^18^O)

Bottom-water temperatures and salinities were reconstructed using paired measurements of the Mg/Ca ratio and δ^18^O on benthic foraminifera shells [68,69]. In addition, the Mn/Ca ratio was used to qualitatively assess variations in the bottom-water oxygen conditions [70,71]. After crushing and cleaning following the procedure described by Barker and colleagues [72], the benthic foraminifera were transferred to an ICP-OES to analyze their elemental composition (for details, see S1 Text). The Mg/Ca values were converted into bottom-water temperatures using well-established calibrations for the species used (see S5 Table). Eliminating the global ice volume impact [40] and the Mg/Ca-based temperature effect from the benthic foraminifera δ^18^O data allows to calculate the seawater δ^18^O (δ^18^O_SW_), a conventional proxy for paleosalinity (e.g., [68]) (for details, see S1 Text). All δ^18^O data have been obtained by analyzing benthic foraminifera (see S5 Table for species used) by mass spectrometry. In addition to previously published data, here, we provide new δ^18^O data for the Moroccan (Gulf of Cádiz) and Mauritanian margins (for details, see S1 Text and S3 Fig).

#### Vertical and lateral food supply based on BFARs and grain-size analyses

As suspension feeders, CWCs are very sensitive to food availability controlled by vertical as well as lateral food supply [18,22,23,25]. The food supply is reconstructed by using the benthic foraminifera accumulation rate (BFAR) expressed as the number of shells cm^−2^ ka^−1^ that is positively related to organic matter fluxes to the sea floor, commonly reflecting export productivity [73,74]. However, slope sites influenced by benthic nepheloid layers may be additionally impacted by lateral organic matter input, which is controlled by the strength of the bottom-water hydrodynamics. Strong bottom currents not only provide an enhanced lateral supply of suspended food particles to the sessile CWCs [23,24], but also cause the deposition of coarser sediments, while finer sediments indicate weaker bottom currents and, consequently, lower lateral food supply [44,75]. The grain-size data of nearly all study sites were published previously (S2 Table). Only for the Mauritanian margin a new grain-size record was produced. Further details of the applied methodologies can be found in S1 Text and in the original references listed in S2 Table.

#### Statistical analyses (NLR)

In order to identify statistically significant environmental predictors for the proliferation of CWCs, we applied a nominal logistic regression (NLR) model to the paleoceanographic proxy data and periods of presence or absence of corals based on the coral age data. The binary *L*. *pertusa* occurrence status (absent = 0, present = 1) was regressed on the paleoenvironmental parameters as predictors. Since not all paleoenvironmental parameters were coregistered with the CWC occurrence data (marked as vertical yellow bars in Figs 2 and 3), we tested for the effects of paleoenvironmental parameters individually. The obtained *p*-Values (S6 Table) for the regressions were corrected for multiple tests applying the Dunn–Sidak correction method [76]. For these straightforward calculations, no specific software was required.

## Results

### The Gulf of Mexico and Irish margin

In the Gulf of Mexico (Campeche Bank) and at the Irish margin (Porcupine Seabight), *L*. *pertusa* growth started at approximately 10 kyr BP and approximately 11kyr BP, respectively (Figs 2 and 3, S7 and S8 Figs), short before the global Holocene Climatic Optimum was reached at approximately 9 kyr BP [77]. At both sites, bottom-water temperature and salinities were somewhat similar before and after the onset of *L*. *pertusa* growth (Fig 2A and 2B). All recorded temperature values (between 5 and 12°C; Fig 2A and 2B) are well within the known tolerance range of *L*. *pertusa* [5,78]. At the Irish margin, a major drop in δ^18^O_sw_ of 1 ‰ suggests significant freshening between 6 and 9 kyr BP, without any impact on the proliferation of the CWCs (Fig 2B). The bottom-water oxygenation (expressed by the Mn/Ca ratios) reveals some changes coinciding with the onset of *L*. *pertusa* growth. In the Gulf of Mexico (Campeche Bank), oxygenation actually decreased with the onset of coral growth, while on the Irish margin (Porcupine Seabight) a slight increase was observed (Fig 3A and 3B). However, on the Irish margin, this was followed by the lowest oxygenation at 8 to 9 kyr BP without any effect on the CWCs (Fig 3B). In both areas, *L*. *pertusa* started to grow at the same time when an increased flux of food reached the seafloor (expressed by higher BFAR), which for the site on the Irish margin is followed approximately 4 kyr later by an even stronger rise (Fig 3A and 3B). The simultaneous enforcement of the bottom-water hydrodynamics (expressed by coarser grain sizes) suggest horizontal food supply as an important factor during this period in both regions (Fig 3A and 3B) [44,49].

### The Moroccan (Gulf of Cádiz) and Mauritanian margins

Along the Moroccan (Gulf of Cádiz) and Mauritanian margins, *L*. *pertusa* experienced a most prolific period during the deglaciation period, while it declined at approximately 11 kyr BP just prior to the Holocene Climatic Optimum (Figs 2 and 3, S9 and S10 Figs) [45,52–54]. However, a gap in coral occurrence overlaps the prolific period in both locations and coincides (although only partially in the Gulf of Cádiz) with the HS1, a millennial-scale paleoclimate event (Figs 2C, 2D, 3C, and 3D). During the last 20 kyr BP, the bottom-water temperatures oscillated between 8 and 14°C on the Moroccan margin (Gulf of Cádiz) and between 8.5 and 10.5°C off Mauritania (Fig 2C and 2D). All of these values are within the range of known *L*. *pertusa* tolerances [5,78]. Both temperature and δ^18^O_sw_ (salinity) did not show any significant changes and/or trends that align with the *L*. *pertusa* demise at the onset of the Holocene, at around 11 kyr BP (Fig 2C and 2D). Nonetheless, on the Mauritanian margin, a sharp increase of δ^18^O_sw_ at 18 kyr BP coincides with a period of coral absence related to the HS1 event (Fig 2D). However, the subsequent reoccurrence of *L*. *pertusa* at 14 kyr BP and its collapse at the onset of the Holocene were not linked to any drastic changes in δ^18^O_sw_ (Fig 2D). Unfortunately, there are no records of temperature, salinity, and oxygenation for the Moroccan margin (Gulf of Cádiz) between 14 and 20 kyr BP (Figs 2C and 3C) due to a change in benthic foraminifera assemblage composition. In general, dissolved oxygen concentrations displayed some variability during the last 20 kyr BP, but as it seems oxygen had no controlling effect on *L*. *pertusa* in both regions (Fig 3C and 3D).

Along the Moroccan margin (Gulf of Cádiz), decreasing mean grain sizes in the course of the deglaciation indicate a weakening of the bottom currents and, thus, a decrease of lateral food supply [52] (Fig 3C and 3D). The organic matter flux to the seafloor was generally low as indicated by BFAR values of < 700 shells cm^−2^ ka^−1^. Enhanced fluxes occurred between approximately 18 and 4 kyr BP (>250 shells cm^−2^ ka^−1^; highest fluxes between approximately 18 and 12 kyr BP with 300 to 700 shells cm^−2^ ka^−1^). An influence on the demise of *L*. *pertusa* at 11 kyr BP could not be detected (Fig 3C). On the Mauritanian margin, a weakening of the bottom currents in the course of the deglaciation was also observed (Fig 3D) well before the demise of *L*. *pertusa*. Although the bottom current strength weakened, the organic matter flux remained high (BFAR > 10,000 shells cm^−2^ kyr^−1^) until 10 kyr BP, which fits well with the occurrence of *L*. *pertusa*. Afterward, the flux declined (BFAR < 9,000 shells cm^−2^ kyr^−1^) and corals were absent (Fig 3D).

### The western Mediterranean Sea (Alboran Sea, West and East Melilla coral mound provinces)

**Fig 4:**
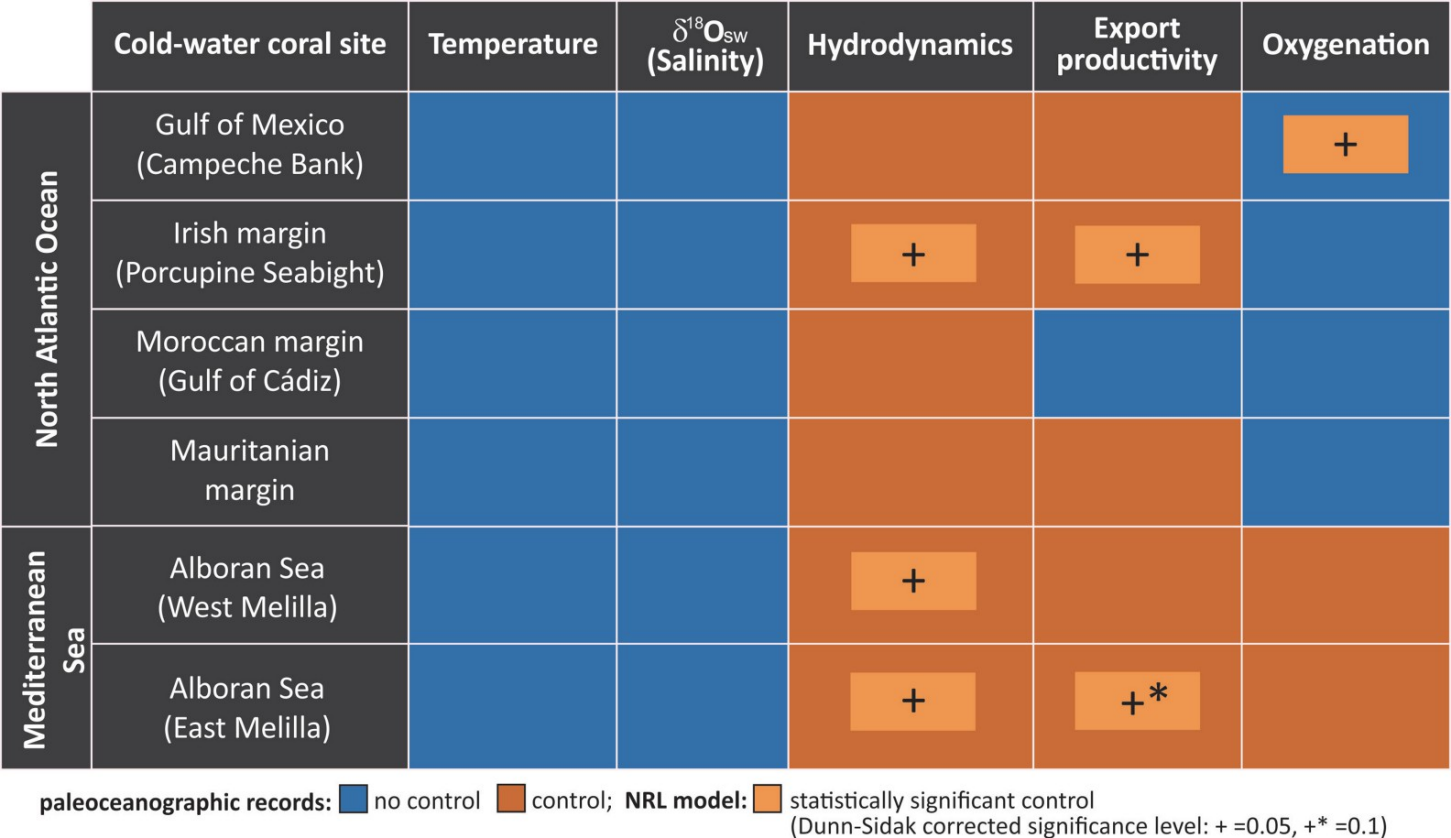
The key environmental driver matrix. Summary of environmental parameters controlling the occurrence of the *Lophelia pertusa* in the North Atlantic and the Mediterranean Sea over the last 20 kyr (blue: no control, orange: control; based on paleoceanographic proxy data). Applying the NLR model to the paleoceanographic records of the selected environmental parameters in combination with records of *L*. *pertusa* occurrence enables us to identify which parameters had the most impact on coral development, i.e., triggering the onset or demise of CWC proliferation. The underlying data for this figure can be found in https://doi.org/10.1594/PANGAEA.932775. NLR, nominal logistic regression.

In the western Mediterranean Sea (Alboran Sea), *L*. *pertusa* was absent during the early deglacial period until it reoccurred at approximately 14 kyr BP (Figs 2 and 3, S11 and S12 Figs) [55,56,61]. The 2 known CWC ecosystems in the West and East Melilla coral mound provinces developed predominantly between approximately 14 kyr BP to 8 to 9 kyr BP [56–58]. The bottom-water temperature was relatively constant in both coral mound provinces (West Melilla: between 9 and 12°C; East Melilla: between 10 and 14°C) during the last 20 kyr, with any significant changes and/or trends that align with the reoccurrence or demise of *L*. *pertusa* (Fig 2E and 2F). The bottom-water δ^18^O_sw_ (salinity) displayed a sharp decrease after 13 kyr BP, although a major shift in δ^18^O_sw_ appears slightly delayed in the East in comparison to the West Melilla province (Fig 2E and 2F), which might be due to an offset in water depth of approximately 100 m between the water depth levels of the 2 off-mound cores. Indeed, *L*. *pertusa* was thriving under high (at approximately 14 kyr BP) as well as under low salinities (at approximately 9 kyr BP) in both locations. Between 8 and 14 kyr BP, an increased BFAR (up to 27,000 shells cm^−2^ ka^−1^) suggests high organic matter flux to the seafloor, while at the same time coarser mean grain sizes (>10 μm) point to strong hydrodynamics enhancing the lateral food supply (Fig 3E and 3F). Both parameters show a slightly different pattern, indicating first the strengthening of the bottom current regime reaching a maximum at approximately 12 kyr BP that was followed by a maximum flux of organic matter reaching the seafloor lasting from 11 kyr BP to 8 to 9 kyr BP (Fig 3E and 3F). Most importantly, low values of BFAR (<7,000 shells cm^−2^ ka^−1^) and finer grain sizes (<10 μm) coincided with the periods of *L*. *pertusa* absence in both locations after 8 to 9 kyr BP (Fig 3E and 3F). Another prominent signal is the decline in oxygenation (increased values of Mn/Ca ratios) after 8 to 9 kyr BP, which also coincides with regional demise of *L*. *pertusa* (Fig 4E and 4F).

## Discussion

### The influence of temperature and salinity

CWCs have a wide thermal tolerance (4 to 15°C) [5,19,78] and are able to thrive in regions with large daily to monthly variations in temperature (up to 9°C) [79]. Due to the continuous ocean warming projected for the next decades and its potential impact on the marine ecosystems [12,15], an increasing number of laboratory experiments have been focusing on the response of CWCs to changes in temperature—especially high temperatures—and the combined effect with other parameters (e.g., *p*CO_2_ and O_2_) [13,30,31,36]. However, these experiments have shown inconclusive results. On geological perspectives, paleoceanographic studies have been pointing to temperature as a main environmental driver for CWC development in the North Atlantic over the most recent geological time [45,80], although bottom-water temperature reconstructions are still rare [47,81]. Controversially, our paleoceanographic results show that the long-term temperature conditions exerted no direct influence on the proliferation or demise of *L*. *pertusa* in the studied locations (Fig 2). The bottom-water temperature at the studied locations in the North Atlantic and the Mediterranean Sea remained relatively stable, showing no short-term oscillations larger than the methodological uncertainty of Mg/Ca ratio-derived temperatures of up to ±1.7°C (depending on the location) over the last 20 kyr (Fig 2), with no increasing trend resembling the 3 to 4°C warming of the surface ocean over the course of the last deglaciation [39,41]. Our results are consistent with temperature reconstructions at mid-depths (300 to 800 m) in the North Atlantic, which also point to relatively constant bottom-water temperatures, e.g., in the Gulf of Mexico [82], at the SW Rockall Trough margin [81] and at the NW African margin [83] over the same period of time. Although millennial-scale temperature oscillations of 2 to 5°C have been recorded at some locations (e.g., Gulf of Mexico and Moroccan margin; Fig 2A and 2C), temperature did not show any significant changes and/or trends that coincide with the on- and offsets of CWC proliferation during the last 20 kyr. This was also documented for the Northwestern Atlantic off Cape Lookout [47]. Even though this CWC-rich region is today affected by large daily-to-monthly temperature variations related to the variability of the Gulf Stream [79], benthic foraminifera Mg/Ca data revealed stable bottom-water temperatures over the past 40 kyr BP, making any strong temperature effect on the coral development off Cape Lookout rather unlikely [47]. All reconstructed absolute temperature values for the North Atlantic and the Mediterranean coral sites ranged between 5 and 15°C (Fig 2) and hence fall within the thermal tolerance of *L*. *pertusa* (4 to 15°C) documented in the literature [5,78]. It suggests that *L*. *pertusa* has not been exposed to temperatures above or below its tolerance at any given time at any study site and temperature may have oscillated within the corals’ tolerance since the last glacial maximum.

CWCs are known to tolerate a wide range of salinities (33 to 39 psu) [5], thus covering almost the complete salinity range found in the world oceans. Nevertheless, salinity has been included in predictive habitat models as a significant parameter on determining a suitable habitat for CWC species [18], although the effect of salinity on CWCs is poorly understood and has never been tested in laboratory experiments [37]. In this context, our paleoceanographic study provides the first insights to the potential impact of salinity on CWC development. Even though δ^18^O_sw_-based salinity reconstructions provide only qualitative salinity information as calibration-based calculation of palaeosalinities suffer from large uncertainties, the relative changes of our δ^18^O_sw_ data clearly indicate that the long-term salinity conditions exerted no direct influence on the on- and offsets of *L*. *pertusa* proliferation in the studied locations (Fig 2). *L*. *pertusa* started to recolonize the western Mediterranean Sea (East and West Melilla) at approximately 14 kyr BP, when high salinity conditions (indicated by high δ^18^O_sw_ values) prevailed and was not affected by a substantial freshening indicated by a 2 to 2.5‰ δ^18^O_sw_ drop that occurred after 13 kyr BP (Fig 2E and 2F). Therefore, it is assumed that salinity did not have a major impact on their reoccurrence and/or demise. On the Irish margin (Porcupine Seabight), *L*. *pertusa* proved to be resilient to strong salinity change suggested by 1.5 ‰ δ^18^O_sw_ drop observed between 6 and 9 kyr BP (Fig 2B). The only possible influence of salinity is recorded from the Mauritanian margin at the beginning of the deglaciation, when a sharp increase in δ^18^O_sw_ suggests a significant salinity increase at the onset of the HS1 event (18 kyr BP) that was accompanied by a local demise of *L*. *pertusa* (Fig 2D).

Finally, our interpretation based on the paleoceanographic records is also supported by the results of the NLR, which clearly showed that temperature and δ^18^O_sw_ as a salinity proxy are not statistically significant predictors for the occurrence of *L*. *pertusa* at all sites studied in the North Atlantic and the Mediterranean Sea (Fig 4).

### The role of bottom-water oxygenation

Low dissolved oxygen concentrations are another critical parameter potentially suppressing the occurrence of CWCs [21,27,29,31] and may pose severe threats for benthic ecosystems as OMZs spread worldwide under ongoing global warming [12,14,15,84]. Laboratory experiments documented that the low oxygenation negatively affects the physiology of CWCs [19,31], as concentrations below 1.5 ml l^−1^ were lethal for *L*. *pertusa* [13]. Interestingly, this conflicts with recent field observations, where small and dispersed living colonies as well as flourishing reefs have been found under hypoxic conditions (0.5 to 1.3 ml l^−1^) within the OMZs off the western African margin [21,27,29,85]. Based on geological records, a temporary absence of CWCs in the eastern Mediterranean during the Early Holocene was interpreted as a local CWC extinction event caused by the anoxic event that culminated into Sapropel layer S1 formation between 6 and 11 kyr BP [86]. In the Atlantic Ocean, events of CWC demise on the Mauritanian margin [54] and off Namibia [87] have been associated with a possible intensification of the regional OMZ, although no direct oxygenation proxies have been applied to support this interpretation.

Here, we provide qualitative estimates of bottom-water oxygenation for all case studies applying benthic foraminifera Mn/Ca ratio. Our results from the Gulf of Mexico (Campeche Bank) actually reveal a decrease in oxygenation when *L*. *pertusa* returned to the region at approximately 10 kyr BP (Fig 3A). The NLR also points to bottom-water oxygenation as a significant predictor in this region (Fig 4). However, as additional evidence based on benthic foraminifera assemblages implies that the regional bottom waters remained relatively well oxygenated also during the Holocene [49], the relative decrease in oxygenation obviously had no negative impact on CWC proliferation in the Gulf of Mexico. On the Irish margin (Porcupine Seabight), the reoccurrence of *L*. *pertusa* at the onset of the Holocene coincided with a slight increase of bottom-water oxygenation (Fig 3B). However, a strong decline in oxygenation between 8 and 9 kyr BP indicated by the highest Mn/Ca ratios over the studied period was insufficient to cause the corals’ mortality (Fig 3B). Hence, oxygen can be ruled out as a main environmental driver for *L*. *pertusa*’s proliferation on the Irish margin. On the Moroccan margin (Gulf of Cádiz), bottom-water oxygenation was relatively stable over the last 14 kyr (the high Mn/Ca value at 12 kyr BP is considered an outlier with no connection with on- and offsets of *L*. *pertusa* proliferation; Fig 3C).

Intriguingly, for the Mauritanian margin, where currently an intense OMZ is assumed to limit the widespread occurrence of CWCs [29,54], our results do not show any link between changes in dissolved oxygen concentrations and the on- and offsets of *L*. *pertusa*’s proliferation over the last 20 kyr (Fig 3D). This may be due to the fact that our oxygen record is derived from a site located within the current range (300 to 700 m) of the local OMZ [84]. Even with ca 120 m rising of the sea level over the last deglaciation [40], our off-mound core as well as the Mauritanian CWC ecosystem (approximately 400 to 600 m) remained within the OMZ. Despite the OMZ setting, the oxygen concentrations may still have been enough to sustain coral growth as observed today on the Angola margin [21,27,85]. Thus, changes observed in the Mn/Ca record may reflect small shifts in bottom-water oxygenation within the hypoxic background that had no governing effect on corals. Our interpretation is further supported by the NLR, which does not indicate any significance of oxygen as a predictor for *L*. *pertusa* on the Mauritanian margin (Fig 4). In contrast, in the western Mediterranean Sea, bottom-water oxygenation was indeed critical for *L*. *pertusa* as a decrease of intermediate water oxygenation at approximately 8 to 9 kyr BP was associated with large-scale mortality of the CWCs in the southern Alboran Sea (Fig 3E and 3F). This partly matches the onset of the Sapropel (S1) layer formation in the East Mediterranean Sea [88], which also caused a temporal extinction of CWCs there [86]. The high sensitivity of *L*. *pertusa* to bottom-water oxygenation in the Mediterranean Sea may be related to the high temperatures of the intermediate waters (up to 14°C) (Fig 3E and 3F), as the oxygen consumption of CWCs increases by 50% with a 2°C increase of temperature [31]. CWCs are well adapted to high temperatures in the Mediterranean Sea [35,37], but the enhanced respiration rate requires high oxygen levels in the surrounding waters [13,31]. Thus, with the declining bottom-water oxygenation at approximately 8 to 9 kyr BP, *L*. *pertusa* was unable to sustain the high metabolism required in these warm waters what probably contributed to their decline in the Alboran Sea. However, the NLR did not detect the bottom-water oxygenation as a significant predictor for *L*. *pertusa* proliferation in the Mediterranean Sea (Fig 4). This is most likely explained by the fact that *L*. *pertusa* was absent during periods of high (between 15 and 20 kyr BP) as well as low (after 8 to 9 kyr BP) bottom-water oxygenation (Fig 3E and 3F). It is noteworthy that the collapse of *L*. *pertusa* in the western Mediterranean (Alboran) Sea was also accompanied by a reduced food supply (Fig 3E and 3F) as will be discussed below.

### Food supply (bottom-water hydrodynamic regimes and export production)

CWCs are sessile suspension feeders and therefore are highly dependent on food supply in their specific location. Thus, they commonly thrive in areas of elevated primary production and/or vigorous bottom-water hydrodynamics [5,22,23,25,89]. Laboratory and field experiments have increasingly documented the importance of food for the physiology of CWCs (e.g., growth and fitness), in particular with respect to their ability to store energy as fatty acids and other nutrients as a survival mechanism for starving periods and maintaining a low metabolism under stressing environmental conditions [21,30,32,35,90–92]. Experimental studies also revealed that the current speed for optimal food capture is approximately 2 to 6 cm s^−1^, while above these values the food capture rate of CWCs is reduced [93,94]. However, as most field observations around thriving CWC reefs measured much higher ambient current velocities (>10 cm s^−1^; [25,95–98] as those experimentally determined, there obviously is a need to study how the coral framework modifies its own local flow environment [99].

Paleoceanographic studies have already related periods of CWC proliferation in the Atlantic Ocean and the Mediterranean Sea with increased surface ocean productivity and vigorous bottom-water currents [43–45,50,57,96]. Here, we complement these studies with new records reconstructing the organic matter flux to the seafloor with the BFAR for all case studies and one new record of bottom-water hydrodynamics (mean grain-size) for the Mauritanian margin. Combining these new with existing data reveals that the on- and offsets of the proliferation of *L*. *pertusa* in the North Atlantic and the Mediterranean Sea were closely associated with food availability either laterally by turbulent bottom-water hydrodynamics and/or vertically by enhanced export production (Fig 3). Comparing these data, the bottom-water hydrodynamic regime appears to be the major supplier of food particles at all sites (Fig 3), partly associated with shifts in water mass configuration, bottom-water currents, and internal wave propagation [25,38,49,54,55,96]. The NLR also points to food supply, either by enhanced export production (Irish margin, Alboran Sea (East Melilla coral mound province)) and/or by intense bottom-water hydrodynamics (Irish margin, both sites in the Alboran Sea) as a significant predictor for the occurrence of *L*. *pertusa* in most of the studied sites in the North Atlantic and the Mediterranean Sea (Fig 4).

Interestingly, our results reveal that CWCs off Mauritania are highly dependent on the export production (Fig 3D). Periods marked by reduced export production (low BFAR), but high bottom-water hydrodynamics (coarse grain sizes), at the Mauritanian margin coincided with the absence of *L*. *pertusa* between 14.7 and 18 kyr BP. In contrast, high BFAR, suggesting enhanced export production, and smaller mean grain-sizes were associated with the presence of *L*. *pertusa* between 14.7 and 10.9 kyr BP (Fig 3D). As discussed before, the occurrence of *L*. *pertusa* along the Mauritanian margin may has been impacted by the OMZ over the last 20 kyr. We argue that, because of the low bottom-water oxygenation, the development of *L*. *pertusa* off Mauritania was only possible, when an elevated amount of fresh and high-quality food was available to compensate such deleterious conditions (Fig 3D). This is supported by recent field observations, suggesting that CWCs are able to tolerate very low oxygen levels of 0.5 to 1.57 ml l^−1^ [21, 29], as long as the energetic costs to sustain vital processes under stressful conditions are compensated by high-quality food availability [21,27]. This possibly enables the CWCs to absorb more energy and, consequently, withstand the existing extreme conditions [32,35]. Consequently, when the flux of organic matter reaching the seafloor, as indicated by the BFAR, declined and the amount of high-quality food was no longer plentiful, corals disappeared from the area during HS1 and with the onset of the Holocene (Fig 3D) and only very recently resettled on the CWC mounds along the Mauritanian margin [54]. Despite the pivotal role of export production on coral survival, none of the investigated environmental parameters was identified by the NLR to be a significant predictor for *L*. *pertusa* off Mauritania (Fig 4).

For the Moroccan margin (Gulf of Cádiz), the long-term occurrence of *L*. *pertusa* also appears to be bound to higher food availability supported by strengthened hydrodynamics, while bottom-water oxygenation shows no pattern related to coral occurrence (Fig 3C). As BFAR stayed high also after the decline of the corals, the hydrodynamics might play the more prominent role in food supply here. This is supported by the by far lowest observed BFAR compared to all other case studies pointing to a generally low export production and food availability in the region, which makes an additional process, hence a strong hydrodynamic regime, delivering food to the corals especially important. Interestingly, the >3 kyr gap in coral occurrence around HS1 has no repercussion in the proxy records and, thus, is difficult to explain. A lack of food supply appears unlikely since both BFAR and bottom-water hydrodynamics were relatively high during this event (Fig 3C), suggesting that another environmental parameter may have played a key role here, which could not be identified with the paleoceanographic proxies applied here. It also means that, irrespective of which environmental changes may have occurred during this period, *L*. *pertusa* was unable to compensate this with food supply. But as the Moroccan margin is characterized by very low export production, the corals might have been quite sensitive to any kind of environmental change. However, as none of the common suspects/proxies point to a significant environmental change off Morocco during this period, it cannot be excluded that the gap in coral occurrence exists only because of the low number of coral datings from this region. None of the investigated environmental parameters were identified by the NLR to be a significant predictor for *L*. *pertusa* off Morocco (Fig 4).

In the western Mediterranean Sea (Alboran Sea), the combined increase of bottom-water hydrodynamics and organic matter supply to the seafloor was clearly associated with the onset of the proliferation of *L*. *pertusa* between 14 and 15 kyr BP (Fig 3E and 3F). However, the decline of mound-forming *L*. *pertusa* reefs to just few living colonies after 8 to 9 kyr BP was not only connected to a reduced food supply, most likely caused by the weak hydrodynamic regime resulting in a reduced lateral food supply, but also to a declined bottom-water oxygenation (Fig 3E and 3F). Our results are consistent with previous studies from the southern Alboran Sea, which also documented a decline in surface productivity, hence in export productivity, as well as poor-ventilated bottom waters due to weak bottom-water hydrodynamics [55–57,100]. Thus, warm and poorly-oxygenated bottom waters associated with reduced food availability point to a deterioration of the living conditions causing the decline of *L*. *pertusa* in the western Mediterranean Sea after 8 to 9 kyr BP (Fig 3E and 3F). This is consistent with the HS1 and the Holocene periods at the Mauritanian margin when *L*. *pertusa* was unable to withstand high temperatures and low oxygenation without food compensation. It confirms that food availability is the dominant environmental parameter controlling the vitality of the CWCs through time, providing energy to maintain their physiological processes (e.g., respiration and calcification). Food is of crucial importance for the corals’ survival especially under the adverse conditions of low oxygen concentrations and/or high temperatures creating a high-energetic demand to compensate for the elevated metabolism. Thus, in all our case studies, climate change–induced modifications in regional productivity and near-bottom hydrodynamic patterns affecting the delivery of food to the corals controlled their past development and may take a decisive role in the future as well.

### Estimating the future fate of CWCs in a changing ocean

Overall, this study offers a basis for further laboratory and modeling experiments in order to understand the response of CWC ecosystems to climate-driven changes, such as the ocean warming and ocean deoxygenation, expected in the coming decades [30,32]. Thus, in order to understand the role of food with respect to compensating the negative effects of these climate-driven changes, the inclusion of food supply (quality and amount) in laboratory experiments should be taken into consideration and evaluated—specifically in combination with additional parameters (e.g., temperature, oxygen concentrations, and *p*CO_2_). This could lead to understanding the role of food in maintaining physiological processes (e.g., respiration and calcification), allowing CWCs to acclimatize and survive under adverse conditions [13,19,30–33,35,36,91]. Experiments manipulating food, temperature, and oxygen concentration would be ideal to test the response of CWCs, for example, to the expansion of the OMZ in times of global warming [12,14,84]. Furthermore, the major outcome of this study is the identification of the crucial role of bottom-water hydrodynamics for the well-being of the corals, which provide the physical energy to sustain the lateral food supply to the corals and appears to be—at least—as important as the vertical food supply triggered by export productivity. Our findings also highlight the importance of predictive modeling simulations to explore regional peculiarities of food transport, such as particulate organic carbon flux to the seafloor (export production) in combination with oceanographic processes such as internal waves, bottom currents, dwelling cascades, and nepheloid layers that control the near-bottom hydrodynamics and, thus, particle transport [101–103]. So far modeling approaches predominantly addressing large geographical scales and using gridded environmental data with relatively coarse spatial resolution (often with a resolution of 1°) found a prominent role of temperature in the distribution of CWCs in the present and in the future ocean [16,18]. Whereas the global spatial distribution of the corals might indeed be strongly controlled by temperature, climate-induced temperature variability in intermediate waters hosting CWC often is in the range of <3°C (Fig 2), which can be even less than present-day short-term (tidal to seasonal) temperature variability [79]. Consequently, for the past and maybe also future, long-term development of CWC in approximately 300 to 800 m water depth temperature does not play a comparable crucial role as for their spatial distribution. Modeling approaches involving hydrodynamic conditions at the seabed on the relevant CWC reef scale where near-bottom hydrodynamics are closely tied to the local seabed topography are by now restricted to regional-to-local scales [104]—due to the need for detailed high-resolution bathymetric and oceanographic data (also including internal tides) and long computation times. Consequently, future modeling studies should focus on smaller geographical scales allowing for the consideration of small-scale processes like the lateral food supply that is one of the most critical control factors for coral reef development. At the end, to enable knowledge-guided management decisions and mitigation policies to protect CWC ecosystems and their services for society [11,17] such small-scale assessments will be inevitable.

## Conclusions

In this study, we went back in time to understand how the ecosystem engineering CWC species *L*. *pertusa* responded to past environmental changes over the last 20 kyr, which correspond to the last major global warming event at the end of the last glaciation in order to gain insights about how this species is likely to respond to future climatic changes. We show that, primarily, changes in lateral and vertical food supply, partly associated with changes in oxygen availability, were the main environmental drivers for the proliferation or absence of *L*. *pertusa* in the North Atlantic and the Mediterranean Sea, whereas temperature and salinity changes hardly had any direct effect. Therefore, we expect that climate-driven changes in oceanic processes on food transport as well as ocean deoxygenation [12,15] are likely to be the determining factors of life and death of CWC species in the coming decades. The intensification of the eastern boundary upwelling systems (e.g., off Mauritania) [105,106] is one of the predicted future scenarios favorable for CWC proliferation. However, the elevated export production induced by intensified upwelling may bring negative feedback by reducing bottom-water oxygenation through the remineralization of the sinking organic matter, possibly hampering the occurrence of CWCs [21,54]. In this scenario, CWCs might survive as a result of the high amount of high-quality food which—as seen in our study—compensates for the low oxygenation [27]. As stated above, future studies should include food as a determining factor to verify its life sustaining influence for CWCs, thereby also considering the important role of near-bottom hydrodynamics for the food supply to the CWCs. On the other hand, the ventilation of the ocean interior and the globally averaged export production are also predicted to decline toward the end of the century in response to enhanced stratification of the upper ocean layers in a warming ocean [12,14,15,84]. The synergy of low food availability and hypoxic conditions would be a catastrophic scenario for CWCs and could endanger their survival. Ocean acidification poses another threat to CWCs as the atmospheric CO_2_ concentration increases constantly [12,15,20], possibly exposing about 70% of the CWCs to corrosive waters by 2100 [107]. Unfortunately, it is challenging to cover the effects of ocean acidification with our paleoceanographic approach as the younger geological history provides no equivalent to the acidic conditions expected at the end of this century [64]. It is difficult to foresee how changes of individual environmental parameters will counterbalance or overlap each other in the future [105]. Over time, it is conceivable that CWCs may be able to regulate their physiology to mitigate the impacts of future changes as long as a sufficient amount of high-quality food is available. However, given the fast speed of the current global climate change, the rate of adaptation might be too slow, which would inevitably lead to mass mortality.

## Supporting information

S1 Fig Age-depth model for core GeoB14885-1 (Mauritanian margin) based on Bacon v. 2.2 [9]. The underlying data for this figure can be found in https://doi.org/10.1594/PANGAEA.932775.
(TIF)

S2 Fig Age model for core GeoB6718-2 from the Irish margin (Porcupine Seabight). The new AMS^14^C ages (S4 Table) were combined with published records of XRF data (Ca(Ca+Fe)) from core GeoB6718-2 and from the nearby core GeoB6719-1, for which also additional AMS ^14^C ages are available [10]. This confirms the age at 108cm core depth of GeoB6718-2 (age in red) as an outlier. Red vertical dashed lines indicate tie-points between both (Ca(Ca+Fe)) curves. The underlying data for this figure can be found in https://doi.org/10.1594/PANGAEA.932775.
(TIF)

S3 Fig **Benthic foraminifera δ^18^O results** from (**A**) core GeoB9064-1 (Moroccan margin, Gulf of Cádiz; measured on *Uvigerina* spp.) and (**B**) core GeoB14885-1 (Mauritanian; measured on *Planulina ariminensis*). The underlying data for this figure can be found in https://doi.org/10.1594/PANGAEA.932775.
(TIF)

S4 Fig Mg/Ca ratios versus Fe/Ca and Al/Ca ratio show low correlation (R^2^ ≤ 0.5) between Mg/Ca and the other ratios for each location, attesting that the cleaning process was efficient and that our elemental/Ca ratios are not affected by any clay contaminations. The underlying data for this figure can be found in https://doi.org/10.1594/PANGAEA.932775.
(TIF)

S5 Fig Temperature with the correspondent Mg/Ca ratios for the 6 locations in the North Atlantic and the Mediterranean Sea over the last 20 kyr BP. (**A**) Gulf of Mexico (Campeche Bank), (**B**) Irish margin (Porcupine Seabight), (**C**) Moroccan margin (Gulf of Cádiz), (**D**) Mauritanian margin, (**E**) Alboran Sea (West Melilla), and (**F**) Alboran Sea (East Melilla). The underlying data for this figure can be found in https://doi.org/10.1594/PANGAEA.932775.
(TIF)

S6 Fig Al/Ca versus Mn/Ca ratios show a low correlation (R^2^ ≤ 0.1) for each location in the North Atlantic and the Mediterranean Sea, indicating that the benthic foraminifera Mn/Ca ratios are not influenced by terrigenous material and consequently reflect redox conditions. The underlying data for this figure can be found in https://doi.org/10.1594/PANGAEA.932775.
(TIF)

S7 Fig The paleoenvironmental history of the Campeche coral mound province in the southern Gulf of Mexico since 20 kyr BP. The occurrence of *Lophelia pertusa* in the region is indicated by black dots at the top (corresponding to published U/Th ages listed in S3 Table). The entire interval of coral growth is highlighted by the yellow vertical bar. The paleoceanographic proxies have been obtained from the off-mound core GeoB16320-2 [36]. (**A**) Bottom-water temperature is based on Mg/Ca ratios (for details, see S4 Table). (**B**) Bottom-water salinity is estimated from δ^18^O_SW_ that was calculated from paired δ^18^O and Mg/Ca measurements (for details, see S4 Table). (**C**) The mean grain-size record is a proxy for the bottom current strength [36]. (**D**) The BFAR, based on foraminifera counting, is a proxy for organic matter flux to the seafloor. (**E**) Mn/Ca ratios measured on *Planulina ariminensis* are a proxy for bottom-water oxygenation (note the inverse axis). Horizontal dashed lines in (A) indicate local modern annual values of temperature at the core site in accordance to WOA2018 [4]. The vertical brown lines define the on- and/or offsets of coral growth. Calibrated AMS ^14^C ages for core GeoB16320-2 are shown as yellow stars at the bottom [36]. The underlying data for this figure can be found in https://doi.org/10.1594/PANGAEA.932775. BFAR, benthic foraminifera accumulation rate.
(TIF)

S8 Fig The paleoenvironmental history of the Porcupine Seabight coral mound province at the Irish margin since 20 kyr BP. The occurrence of *Lophelia pertusa* in the region is indicated by black dots at the top (corresponding to published U/Th ages listed in S3 Table). The entire interval of coral growth is highlighted by the yellow vertical bar. The paleoceanographic proxies have been obtained from the off-mound core GeoB6718-2 [10]. (**A**) Bottom-water temperature is based on Mg/Ca ratios (for details, see S4 Table). (**B**) Bottom-water salinity is estimated from δ^18^O_SW_ that was calculated from paired δ^18^O and Mg/Ca measurements (for details, see S4 Table). (**C**) The mean grain-size record as a proxy for the bottom current strength [10]. (**D**) The BFAR, based on foraminifera counting, is a proxy for organic matter flux to the seafloor. (**E**) Mn/Ca ratio measured on *Cibicides* spp. are a proxy for bottom-water oxygenation (note the inverse axis). Horizontal dashed lines in (A) indicate local modern annual values of temperature at the core site in accordance to WOA2018 [4]. The vertical brown lines define the on- and/or offsets of coral growth. Calibrated AMS ^14^C ages for core GeoB6718-2 are shown as yellow stars at the bottom while the outlier age is shown as red star (this study; see S4 Table and [10]). The underlying data for this figure can be found in https://doi.org/10.1594/PANGAEA.932775. BFAR, benthic foraminifera accumulation rate.
(TIF)

S9 Fig The paleoenvironmental history of the Gulf of Cádiz coral mound province at the Moroccan margin since 20 kyr BP. The occurrence of *Lophelia pertusa* in the region is indicated by black dots (corresponding to U/Th ages listed in S3 Table). The entire interval of coral growth is highlighted by the yellow vertical bar. The paleoceanographic proxies have been obtained from the off-mound core GeoB9064 [37]. (**A**) Bottom-water temperature is based on Mg/Ca ratios (for details, see S4 Table). (**B**) Bottom-water salinity is estimated from δ^18^O_SW_ that was calculated from paired δ^18^O and Mg/Ca measurements (for details, see S4 Table). (**C**) The mean grain-size record as a proxy for the bottom current strength [37]. (**D**) The BFAR, based on foraminifera counting, is a proxy for organic matter flux to the seafloor. (**E**) Mn/Ca ratios measured on *Uvigerina* spp. are a proxy for bottom-water oxygenation (note the inverse axis). Horizontal dashed lines in (A) indicate local modern annual values of temperature at the core site in accordance to WOA2018 [4]. The vertical brown lines define the on- and/or offsets of coral growth. Calibrated AMS ^14^C ages for core GeoB9064 are shown as yellow stars at the bottom [37,38]. The underlying data for this figure can be found in https://doi.org/10.1594/PANGAEA.932775. BFAR, benthic foraminifera accumulation rate.
(TIF)

S10 Fig The paleoenvironmental history of the Mauritanian coral mound province since 20 kyr BP. The occurrence of *Lophelia pertusa* in the region is indicated by black dots at the top (corresponding to U/Th ages listed in S3 Table). The paleoceanographic proxies have been obtained from the off-mound core GeoB14885-1. (**A**) Bottom-water temperature is based on Mg/Ca ratios (for details, see Table D in S1 Text). (**B**) Bottom-water salinity is estimated from δ^18^O_SW_ that was calculated from paired δ^18^O and Mg/Ca measurements (for details, see S4 Table). (**C**) The mean grain-size record is a proxy for the bottom current strength. (**D**) The BFAR, based on foraminifera counting, is a proxy for organic matter flux to the seafloor. (**E**) Mn/Ca ratios measured on *Planulina ariminensis* are a proxy for bottom-water oxygenation (note the inverse axis). Horizontal dashed lines in (A) indicate local modern annual values of temperature at the core site in accordance to WOA2018 [4]. The vertical brown lines define the on- and/or offsets of coral growth. Calibrated AMS ^14^C ages for core GeoB14885-1 are shown as yellow stars at the bottom while the inversion age is shown as red star (this study; S4 Table). The underlying data for this figure can be found in https://doi.org/10.1594/PANGAEA.932775. BFAR, benthic foraminifera accumulation rate.
(TIF)

S11 Fig The paleoenvironmental history of the West Melilla coral mound province in the Alboran Sea (Western Mediterranean) since 20 kyr BP. The occurrence of *Lophelia pertusa* in the region is indicated by black dots at the top (corresponding to AMS ^14^C and U/Th ages listed in S3 Table). The entire interval of coral growth is highlighted by the yellow vertical bar. The paleoceanographic proxies have been obtained from the off-mound core GeoB18131-1 [39]. (**A**) Bottom-water temperature is based on Mg/Ca ratios (for details, see S4 Table). (**B**) Bottom-water salinity is estimated from δ^18^O_SW_ that was calculated from paired δ^18^O and Mg/Ca measurements (for details, see S4 Table). (**C**) The mean grain-size record as a proxy for the bottom current strength [39]. (**D**) The BFAR, based on foraminifera counting, is a proxy for organic matter flux to the seafloor. (**E**) Mn/Ca ratios measured on *Cibicidoides mundulus* are a proxy for bottom-water oxygenation (note the inverse axis). Horizontal dashed lines in (A) indicate local modern annual values of temperature at the core site in accordance to WOA2018 [4]. The vertical brown lines define the on- and/or offsets of coral growth. Calibrated AMS ^14^C ages for core GeoB18131-1 are shown as yellow stars at the bottom [39]. The underlying data for this figure can be found in https://doi.org/10.1594/PANGAEA.932775. BFAR, benthic foraminifera accumulation rate.
(TIF)

S12 Fig The paleoenvironmental history of the East Melilla coral mound province in the Alboran Sea (Western Mediterranean) since 20 kyr BP. The occurrence of *Lophelia pertusa* in the region is indicated by black dots at the top (corresponding to AMS ^14^C and U/Th ages listed in S3 Table). The entire interval of coral growth is highlighted by the light yellow vertical bar. Paleoceanographic proxies have been obtained from off-mound core GeoB1373-1 [40]. (**A**) Bottom-water temperature is based on the Mg/Ca ratios (for details, see S4 Table). (**B**) Bottom-water salinity is estimated from δ^18^O_SW_ that was calculated from paired δ^18^O and Mg/Ca measurements (for details, see S4 Table). (**C**) The mean grain-size record as a proxy for the bottom current strength [40]. (**D**) The BFAR, based on foraminifera counting, is a proxy for organic matter flux to the seafloor. (**E**) Mn/Ca ratios measured on *Cibicidoides mundulus* are a proxy for bottom-water oxygenation (note the inverse axis). Horizontal dashed lines in (A) indicate local modern annual values of temperature at the core site in accordance to WOA2018 [4]. The vertical brown lines define the on- and/or offsets of coral growth. Calibrated AMS ^14^C ages for core GeoB1373-1 are shown as yellow stars at the bottom [40]. The underlying data for this figure can be found in https://doi.org/10.1594/PANGAEA.932775. BFAR, benthic foraminifera accumulation rate.
(TIF)

S1 Table Overview about the 6 case study regions representing CWC provinces in the North Atlantic Ocean and the Mediterranean Sea and the off-mound sediment cores used for the paleoenvironmental reconstructions. Further information are provided in the references listed to the right. CWC, cold-water coral.
(DOCX)

S2 Table List of new (this study) and previously published proxy datasets obtained for the off-mound cores presented in this manuscript. (DOCX)

S3 Table Metadata of published AMS ^14^C and U/Th dates obtained from fossil fragments of the CWC *Lophelia pertusa*. Coral fragments were collected from 6 coral mound provinces in the North Atlantic and the Mediterranean Sea. Only ages encompassing the last 20 kyr are listed and discussed for our study. AMS ^14^C coral ages obtained for the Irish margin and the Mediterranean Sea were recalibrated using the CALIB8.2 software [41]. For the calibration of the Irish coral ages, we have applied the MARINE20 calibration curve [42] with a local reservoir age correction of ΔR = −70 ± 50 years accounting for a marine reservoir age of R = 480 ± 120 years for the Holocene, which is based on paired AMS^14^C –U/Th dating published in Frank and colleagues [43,45]. For the western Mediterranean coral ages, we have applied the MARINE20 calibration curve with a local reservoir age correction of ΔR = −90 ± 80 years accounting for a deglacial to Holocene marine reservoir age of R = 370 ±40 years (according to Reimer and McCormac [46] and Siani and colleagues [47,48]). CWC, cold-water coral.
(DOCX)

S4 Table Raw AMS ^14^C data and calibrated ages of sediment cores GeoB14885-1 and GeoB6718-2. The underlying data for this figure can be found in https://doi.org/10.1594/PANGAEA.932775.
(DOCX)

S5 Table List of benthic foraminifera species-specific equations to estimate BWT and δ^18^O of seawater (δ^18^O_SW_) (δ^18^O_C_ refers to δ^18^O measured on the foraminifera carbonate shells) as well as BWS. The number in the left column refers to the regions presented in Fig 1 to which the respective equations have been applied. References: Shackleton [60]–(SK74), Cacho and colleagues [17]–(CA06), Bryan and Marchitto [13]–(BM08), Huang and colleagues [15]–(HU12), and Marchitto and colleagues [19]–(MA14). BWS, bottom-water paleosalinity; BWT, bottom-water paleotemperature.
(DOCX)

S6 Table *p*-Values of the NLR analyses. Values marked with a superscripted asterisk denote significance at a Dunn–Sidak corrected significance level of 0.05, and superscripted plus denotes significance at a Dunn–Sidak corrected significance level of 0.1. The underlying data for this figure can be found in https://doi.org/10.1594/PANGAEA.932775. NLR, nominal logistic regression.
(DOCX)

S1 Text Supporting information text. (DOCX)

## Abbreviations

BFAR: benthic foraminifera accumulation rate
BP: before present
CWC: cold-water coral
EP: export production
HS1: Heinrich Stadial 1
O_2_: Oxygen
OMZ: oxygen minimum zone
MPA: marine protected area
NLR: nominal logistic regression
