# Supplementary figures and images for: Major environmental drivers determining life and death of cold-water corals through time
Source: PLoS Biol. 2022 May 19;20(5):e3001628. doi: 10.1371/journal.pbio.3001628 (PMC9119455; doi:10.1371/journal.pbio.3001628)

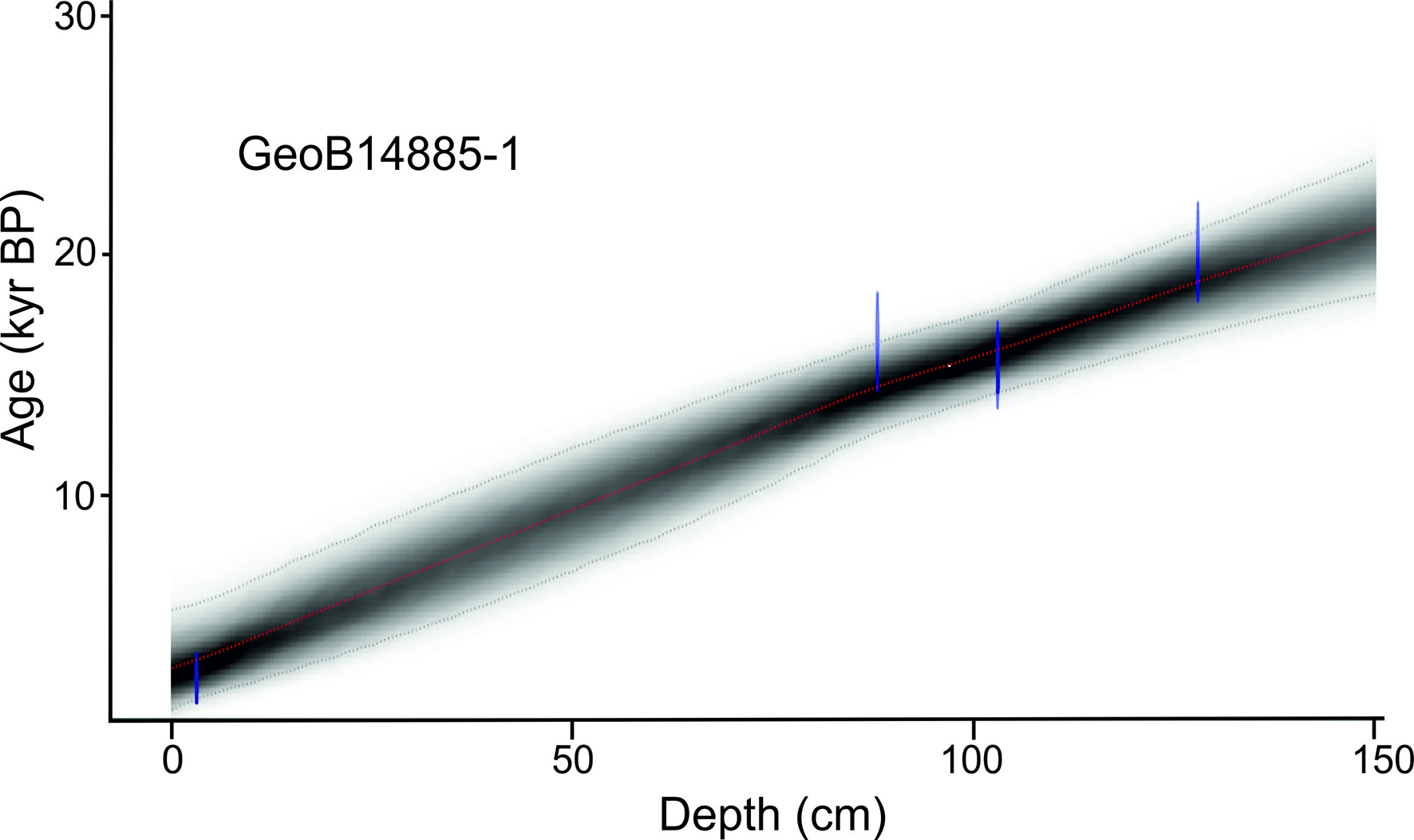

Supplement: S1 Fig — The underlying data for this figure can be found in https://doi.org/10.1594/PANGAEA.932775. (TIF) [file pbio.3001628.s001.tif]

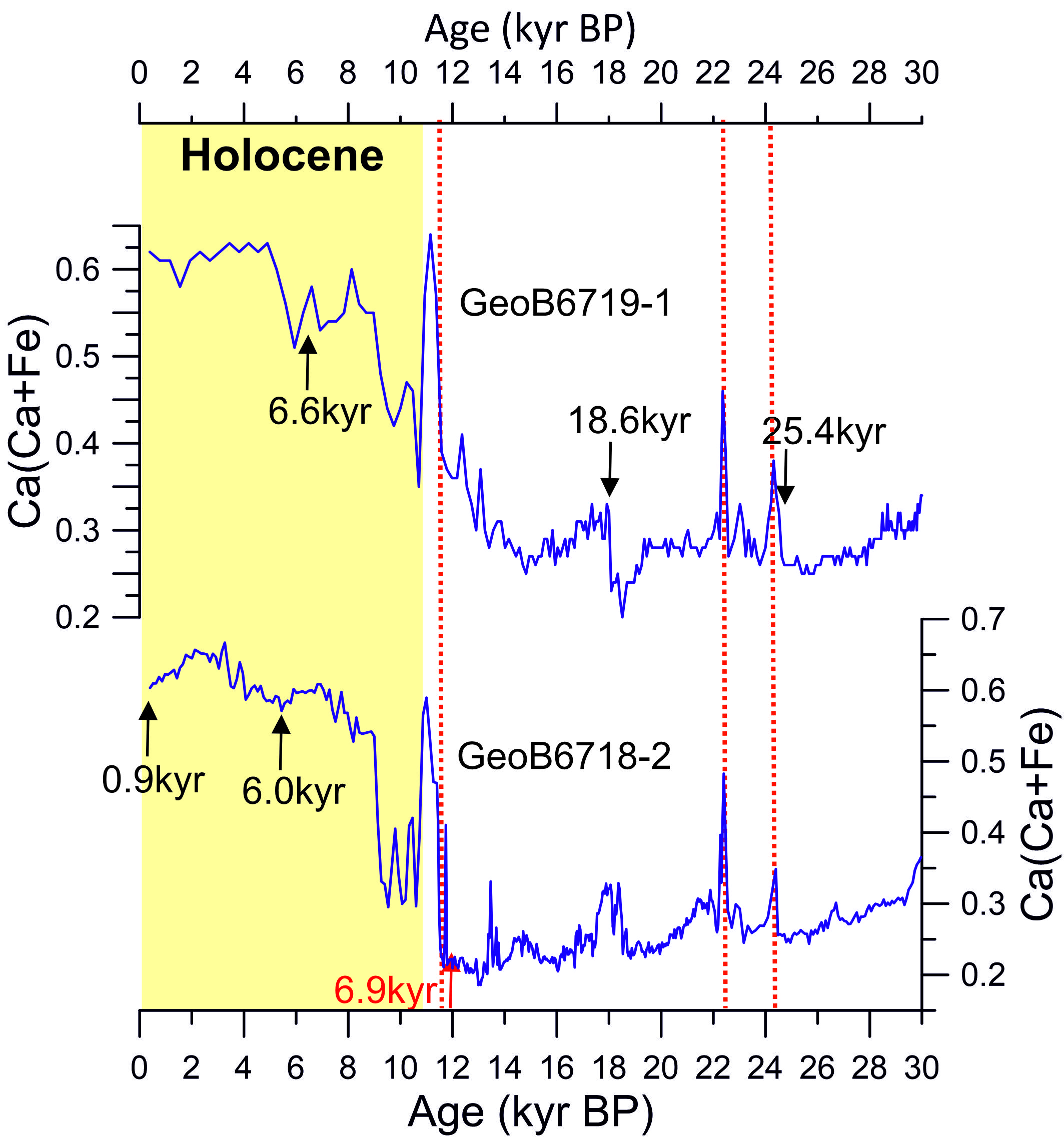

Supplement: S2 Fig — The new AMS14C ages (S4 Table) were combined with published records of XRF data (Ca(Ca+Fe)) from core GeoB6718-2 and from the nearby core GeoB6719-1, for which also additional AMS 14C ages are available [10]. This confirms the age at 108cm core depth of GeoB6718-2 (age in red) as an outlier. Red vertical dashed lines indicate tie-points between both (Ca(Ca+Fe)) curves. The underlying data for this figure can be found in https://doi.org/10.1594/PANGAEA.932775. (TIF) [file pbio.3001628.s002.tif]

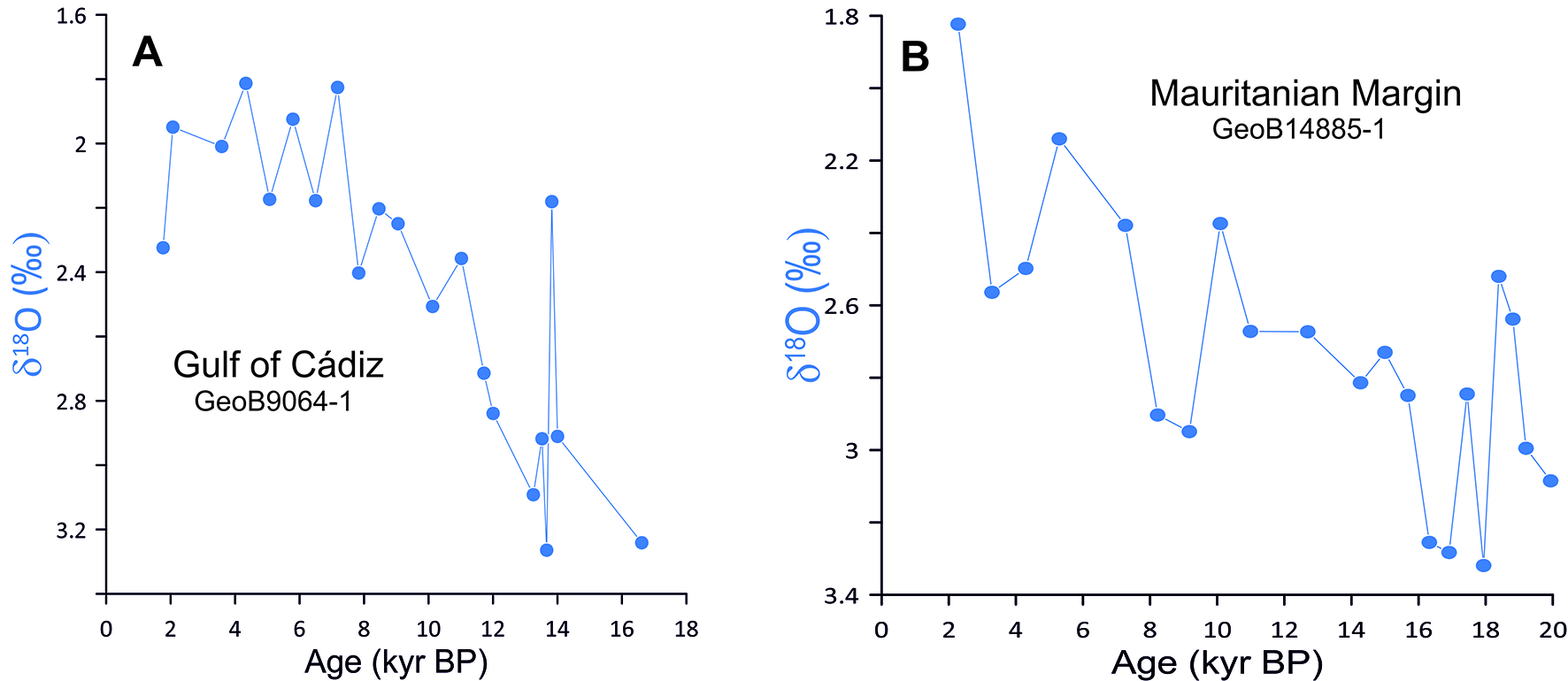

Supplement: S3 Fig — Benthic foraminifera δ18O results from (A) core GeoB9064-1 (Moroccan margin, Gulf of Cádiz; measured on Uvigerina spp.) and (B) core GeoB14885-1 (Mauritanian; measured on Planulina ariminensis). The underlying data for this figure can be found in https://doi.org/10.1594/PANGAEA.932775. (TIF) [file pbio.3001628.s003.tif]

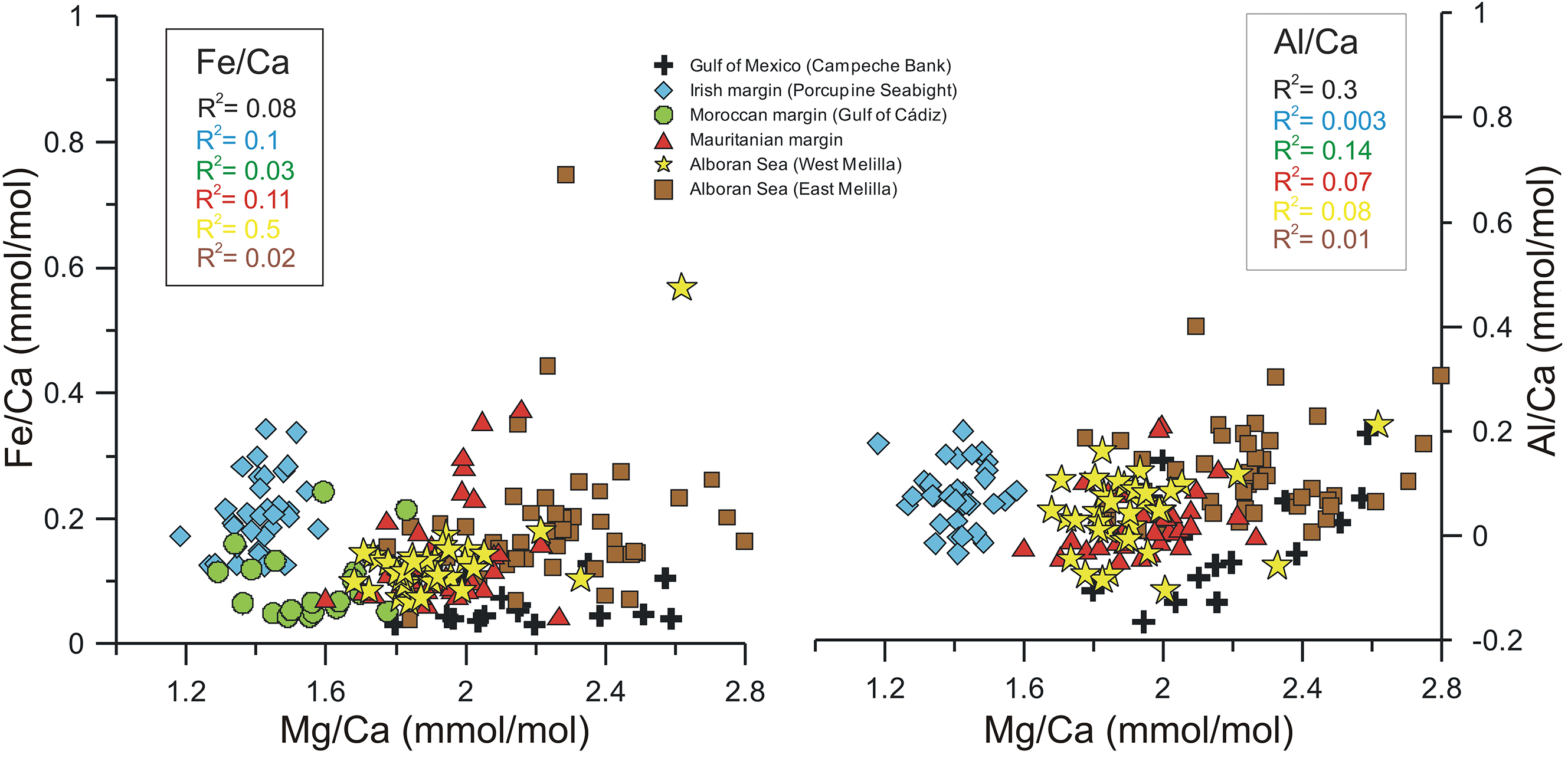

Supplement: S4 Fig — The underlying data for this figure can be found in https://doi.org/10.1594/PANGAEA.932775. (TIF) [file pbio.3001628.s004.tif]

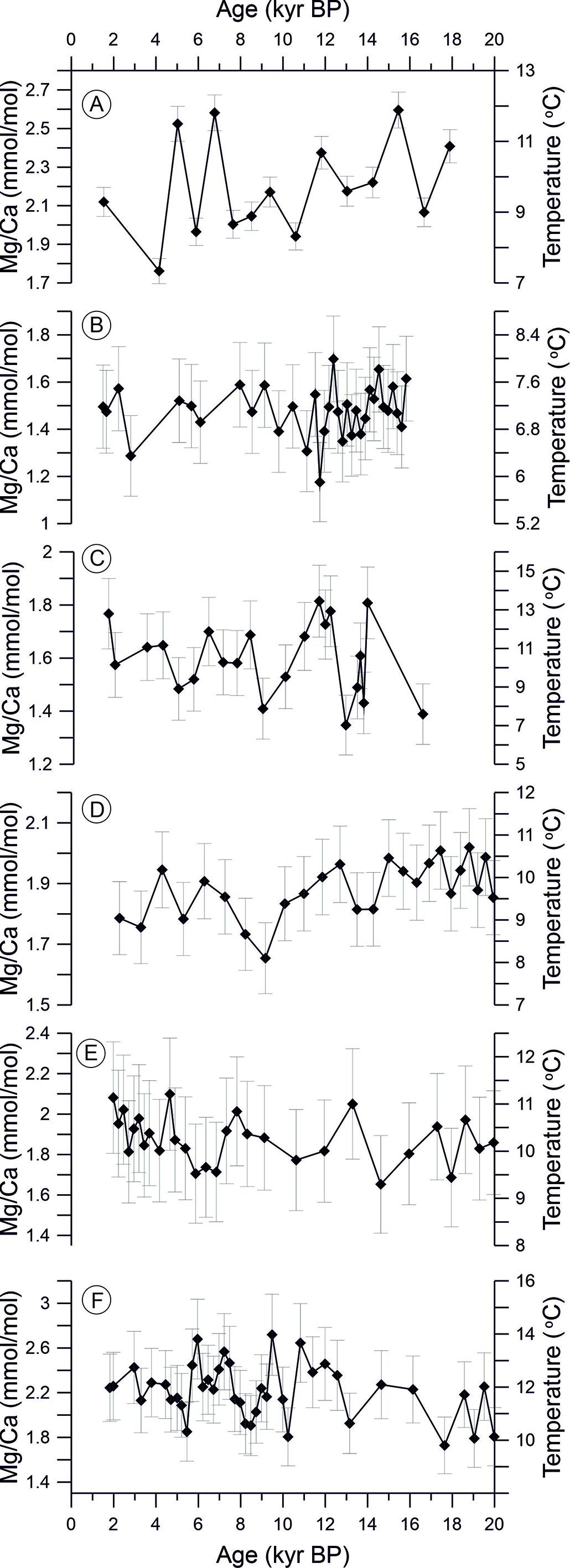

Supplement: S5 Fig — (A) Gulf of Mexico (Campeche Bank), (B) Irish margin (Porcupine Seabight), (C) Moroccan margin (Gulf of Cádiz), (D) Mauritanian margin, (E) Alboran Sea (West Melilla), and (F) Alboran Sea (East Melilla). The underlying data for this figure can be found in https://doi.org/10.1594/PANGAEA.932775. (TIF) [file pbio.3001628.s005.tif]

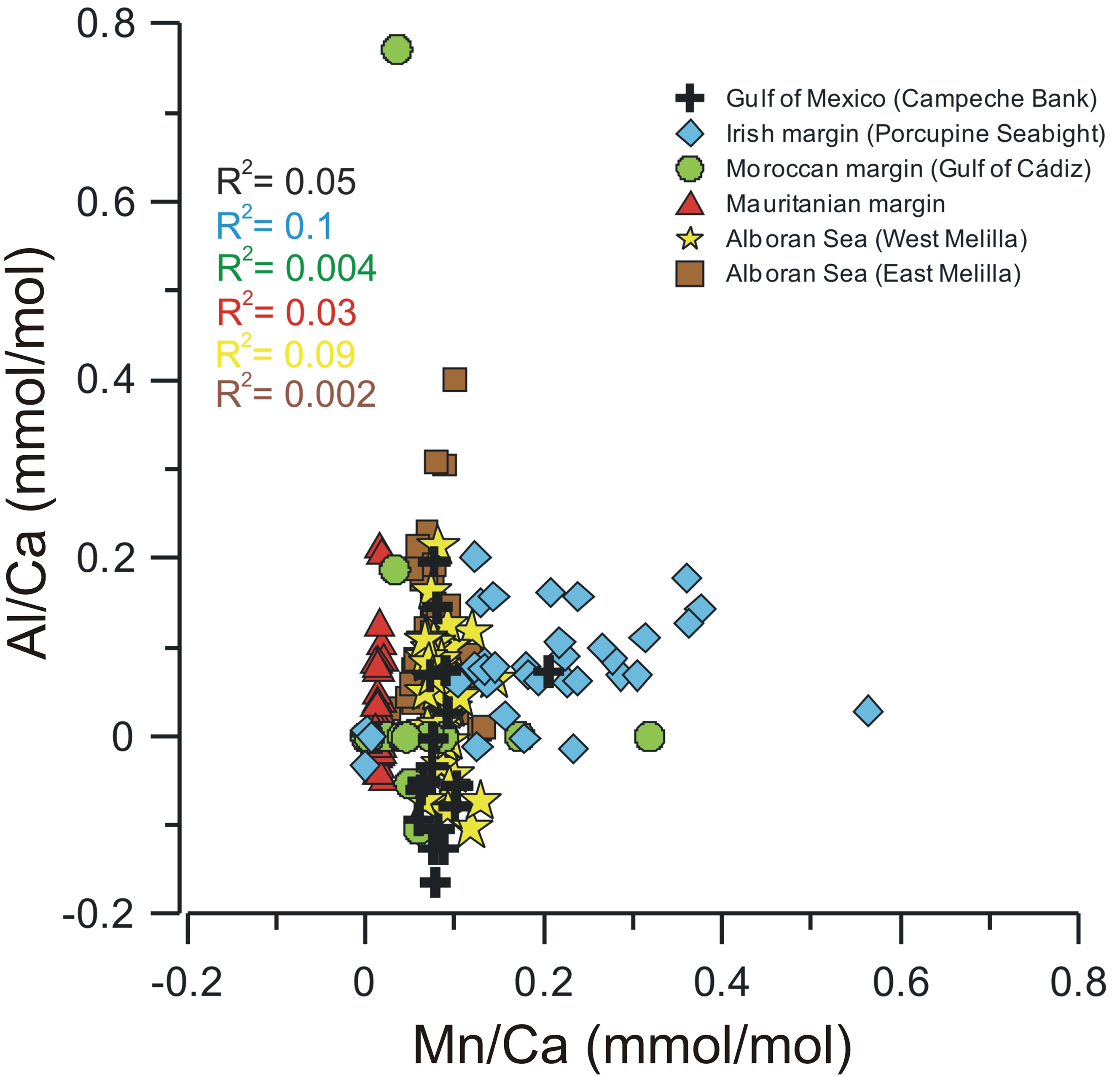

Supplement: S6 Fig — The underlying data for this figure can be found in https://doi.org/10.1594/PANGAEA.932775. (TIF) [file pbio.3001628.s006.tif]

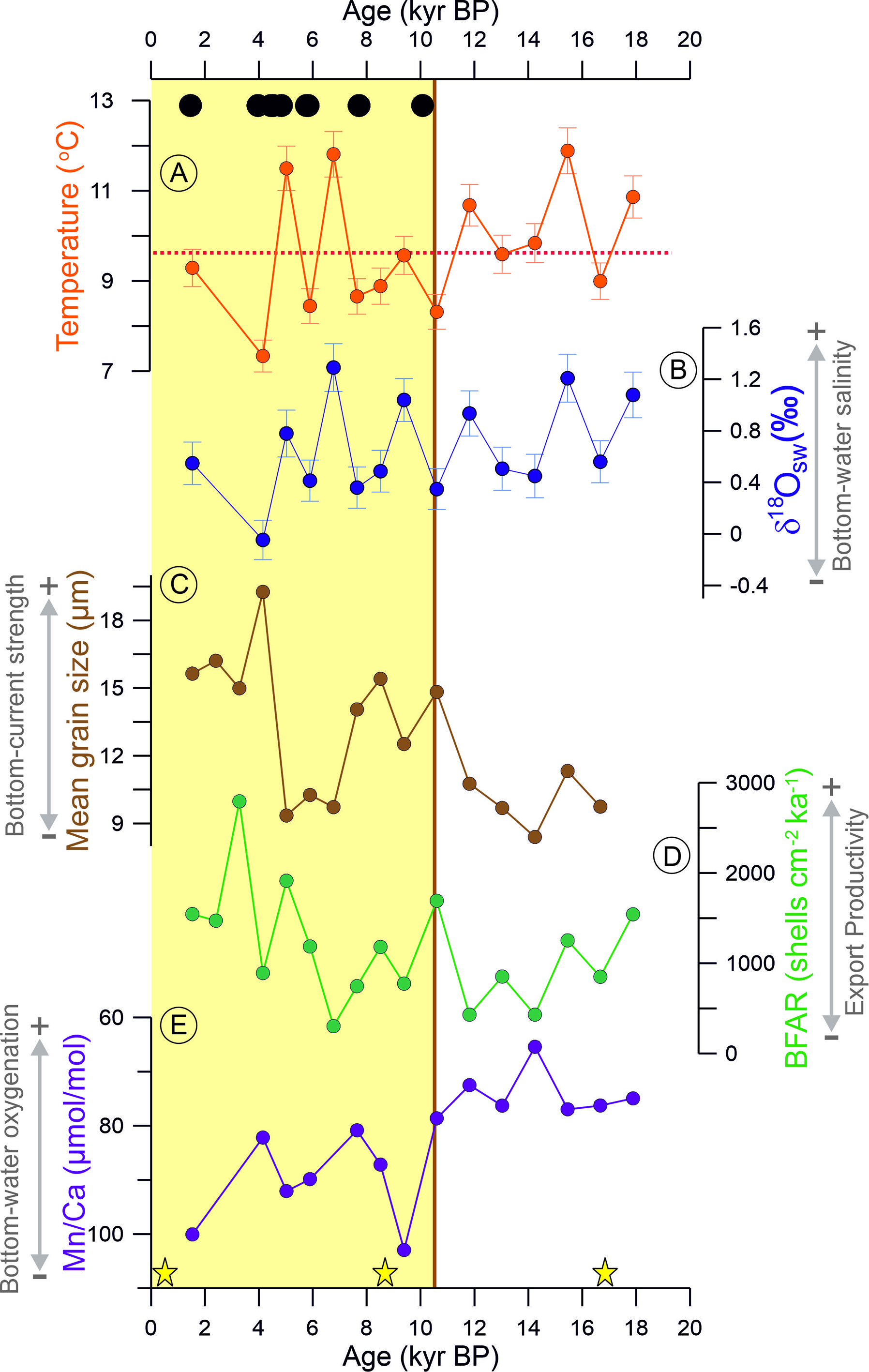

Supplement: S7 Fig — The occurrence of Lophelia pertusa in the region is indicated by black dots at the top (corresponding to published U/Th ages listed in S3 Table). The entire interval of coral growth is highlighted by the yellow vertical bar. The paleoceanographic proxies have been obtained from the off-mound core GeoB16320-2 [36]. (A) Bottom-water temperature is based on Mg/Ca ratios (for details, see S4 Table). (B) Bottom-water salinity is estimated from δ18OSW that was calculated from paired δ18O and Mg/Ca measurements (for details, see S4 Table). (C) The mean grain-size record is a proxy for the bottom current strength [36]. (D) The BFAR, based on foraminifera counting, is a proxy for organic matter flux to the seafloor. (E) Mn/Ca ratios measured on Planulina ariminensis are a proxy for bottom-water oxygenation (note the inverse axis). Horizontal dashed lines in (A) indicate local modern annual values of temperature at the core site in accordance to WOA2018 [4]. The vertical brown lines define the on- and/or offsets of coral growth. Calibrated AMS 14C ages for core GeoB16320-2 are shown as yellow stars at the bottom [36]. The underlying data for this figure can be found in https://doi.org/10.1594/PANGAEA.932775. BFAR, benthic foraminifera accumulation rate. (TIF) [file pbio.3001628.s007.tif]

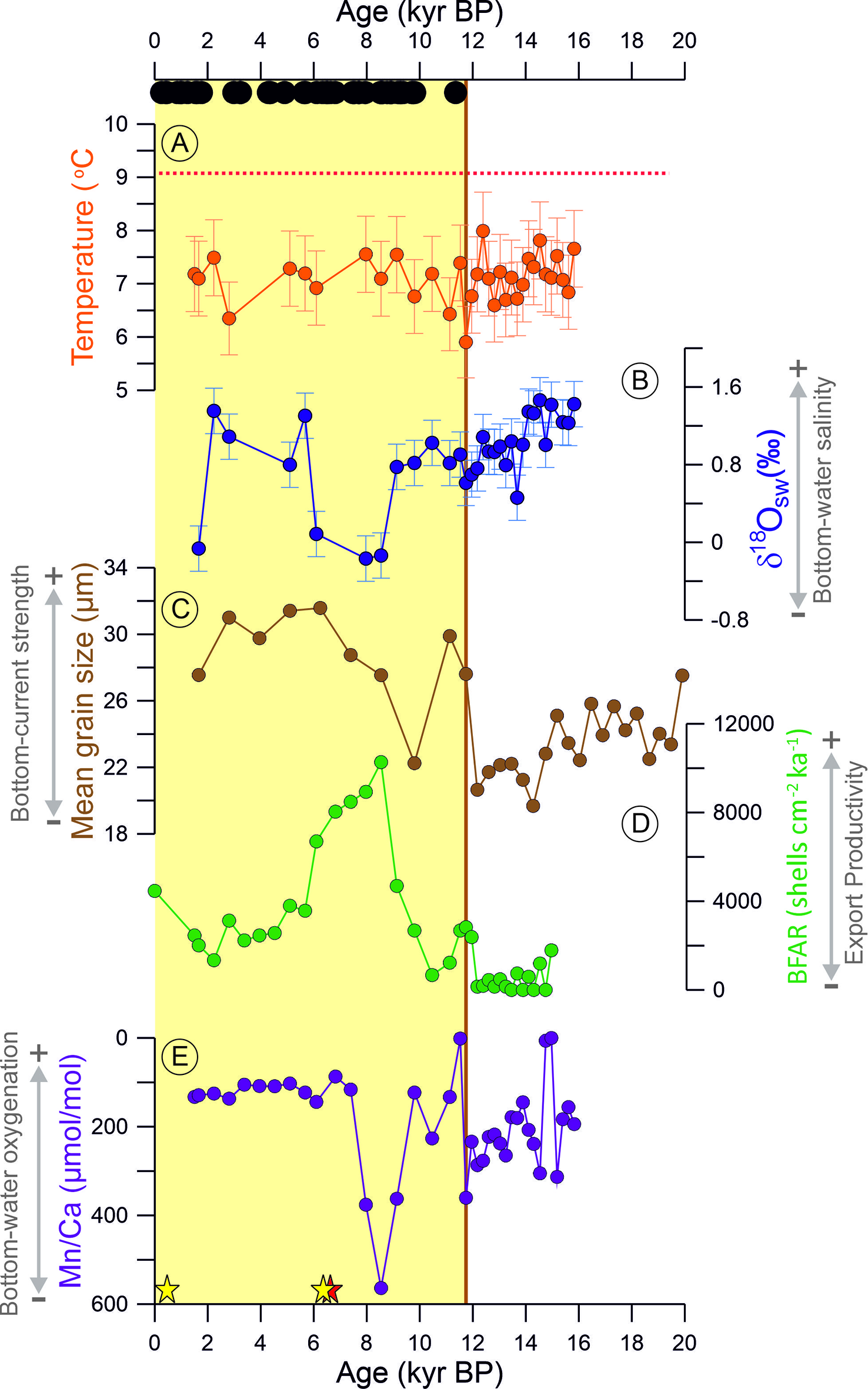

Supplement: S8 Fig — The occurrence of Lophelia pertusa in the region is indicated by black dots at the top (corresponding to published U/Th ages listed in S3 Table). The entire interval of coral growth is highlighted by the yellow vertical bar. The paleoceanographic proxies have been obtained from the off-mound core GeoB6718-2 [10]. (A) Bottom-water temperature is based on Mg/Ca ratios (for details, see S4 Table). (B) Bottom-water salinity is estimated from δ18OSW that was calculated from paired δ18O and Mg/Ca measurements (for details, see S4 Table). (C) The mean grain-size record as a proxy for the bottom current strength [10]. (D) The BFAR, based on foraminifera counting, is a proxy for organic matter flux to the seafloor. (E) Mn/Ca ratio measured on Cibicides spp. are a proxy for bottom-water oxygenation (note the inverse axis). Horizontal dashed lines in (A) indicate local modern annual values of temperature at the core site in accordance to WOA2018 [4]. The vertical brown lines define the on- and/or offsets of coral growth. Calibrated AMS 14C ages for core GeoB6718-2 are shown as yellow stars at the bottom while the outlier age is shown as red star (this study; see S4 Table and [10]). The underlying data for this figure can be found in https://doi.org/10.1594/PANGAEA.932775. BFAR, benthic foraminifera accumulation rate. (TIF) [file pbio.3001628.s008.tif]

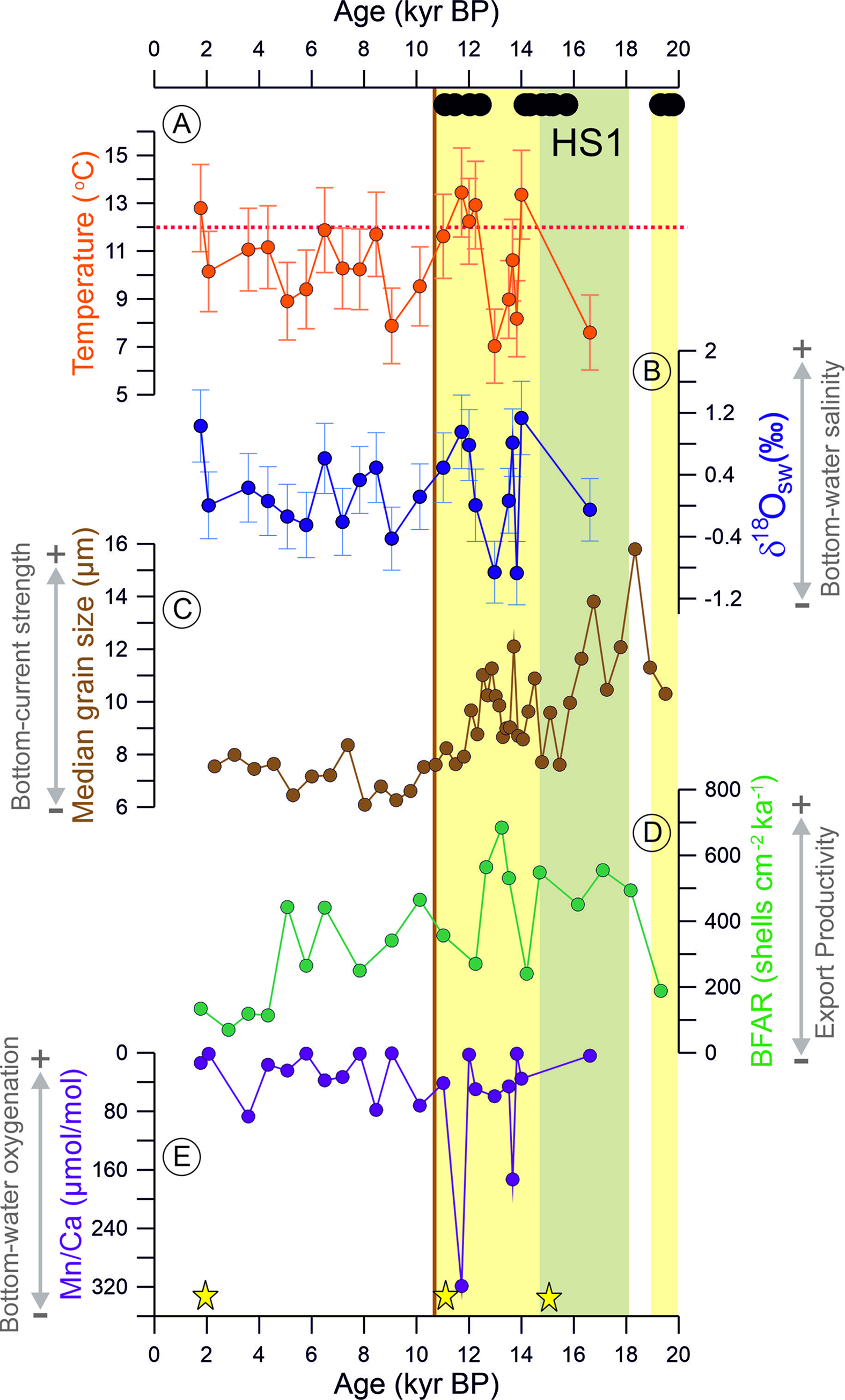

Supplement: S9 Fig — The occurrence of Lophelia pertusa in the region is indicated by black dots (corresponding to U/Th ages listed in S3 Table). The entire interval of coral growth is highlighted by the yellow vertical bar. The paleoceanographic proxies have been obtained from the off-mound core GeoB9064 [37]. (A) Bottom-water temperature is based on Mg/Ca ratios (for details, see S4 Table). (B) Bottom-water salinity is estimated from δ18OSW that was calculated from paired δ18O and Mg/Ca measurements (for details, see S4 Table). (C) The mean grain-size record as a proxy for the bottom current strength [37]. (D) The BFAR, based on foraminifera counting, is a proxy for organic matter flux to the seafloor. (E) Mn/Ca ratios measured on Uvigerina spp. are a proxy for bottom-water oxygenation (note the inverse axis). Horizontal dashed lines in (A) indicate local modern annual values of temperature at the core site in accordance to WOA2018 [4]. The vertical brown lines define the on- and/or offsets of coral growth. Calibrated AMS 14C ages for core GeoB9064 are shown as yellow stars at the bottom [37,38]. The underlying data for this figure can be found in https://doi.org/10.1594/PANGAEA.932775. BFAR, benthic foraminifera accumulation rate. (TIF) [file pbio.3001628.s009.tif]

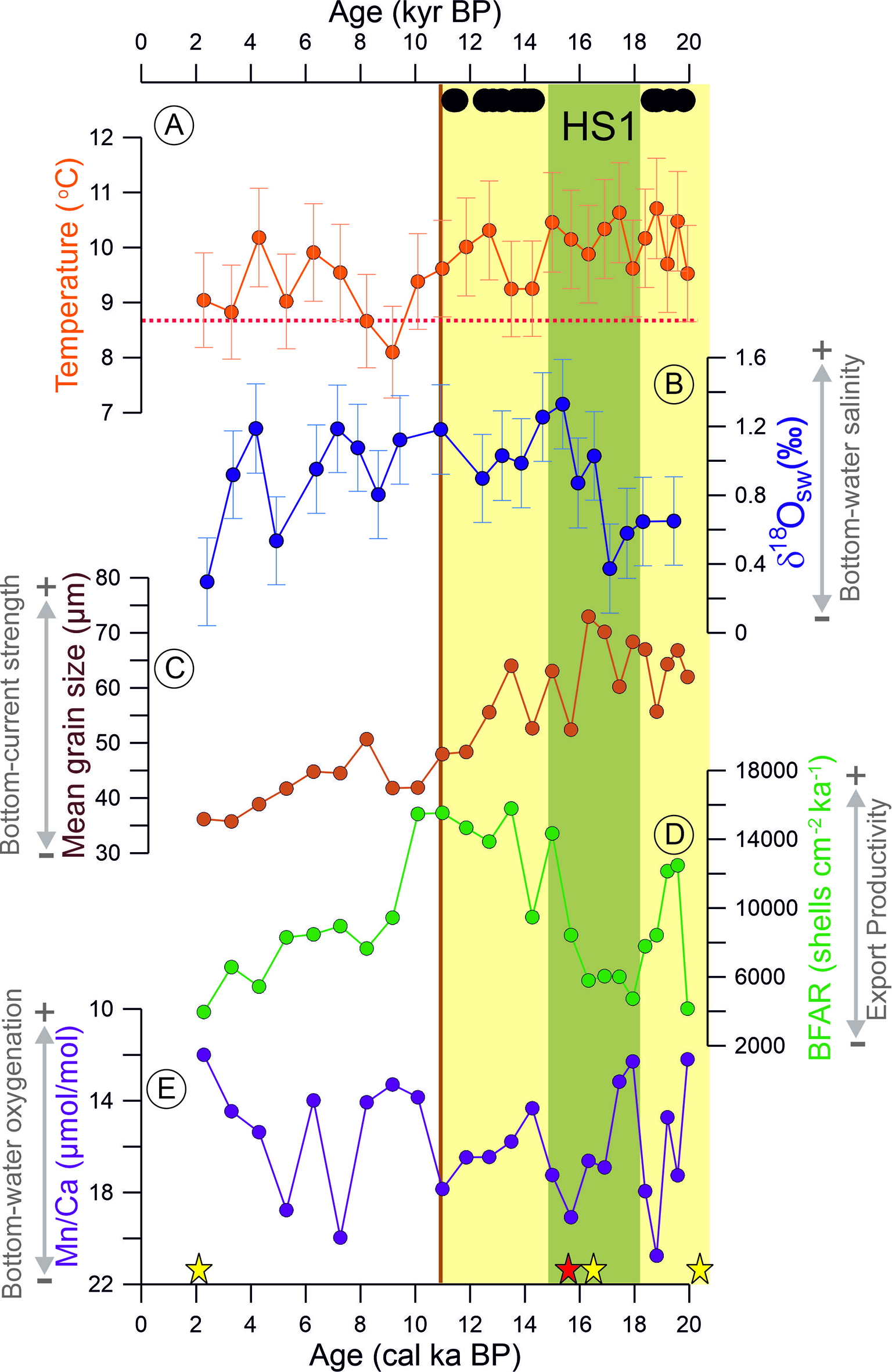

Supplement: S10 Fig — The occurrence of Lophelia pertusa in the region is indicated by black dots at the top (corresponding to U/Th ages listed in S3 Table). The paleoceanographic proxies have been obtained from the off-mound core GeoB14885-1. (A) Bottom-water temperature is based on Mg/Ca ratios (for details, see Table D in S1 Text). (B) Bottom-water salinity is estimated from δ18OSW that was calculated from paired δ18O and Mg/Ca measurements (for details, see S4 Table). (C) The mean grain-size record is a proxy for the bottom current strength. (D) The BFAR, based on foraminifera counting, is a proxy for organic matter flux to the seafloor. (E) Mn/Ca ratios measured on Planulina ariminensis are a proxy for bottom-water oxygenation (note the inverse axis). Horizontal dashed lines in (A) indicate local modern annual values of temperature at the core site in accordance to WOA2018 [4]. The vertical brown lines define the on- and/or offsets of coral growth. Calibrated AMS 14C ages for core GeoB14885-1 are shown as yellow stars at the bottom while the inversion age is shown as red star (this study; S4 Table). The underlying data for this figure can be found in https://doi.org/10.1594/PANGAEA.932775. BFAR, benthic foraminifera accumulation rate. (TIF) [file pbio.3001628.s010.tif]

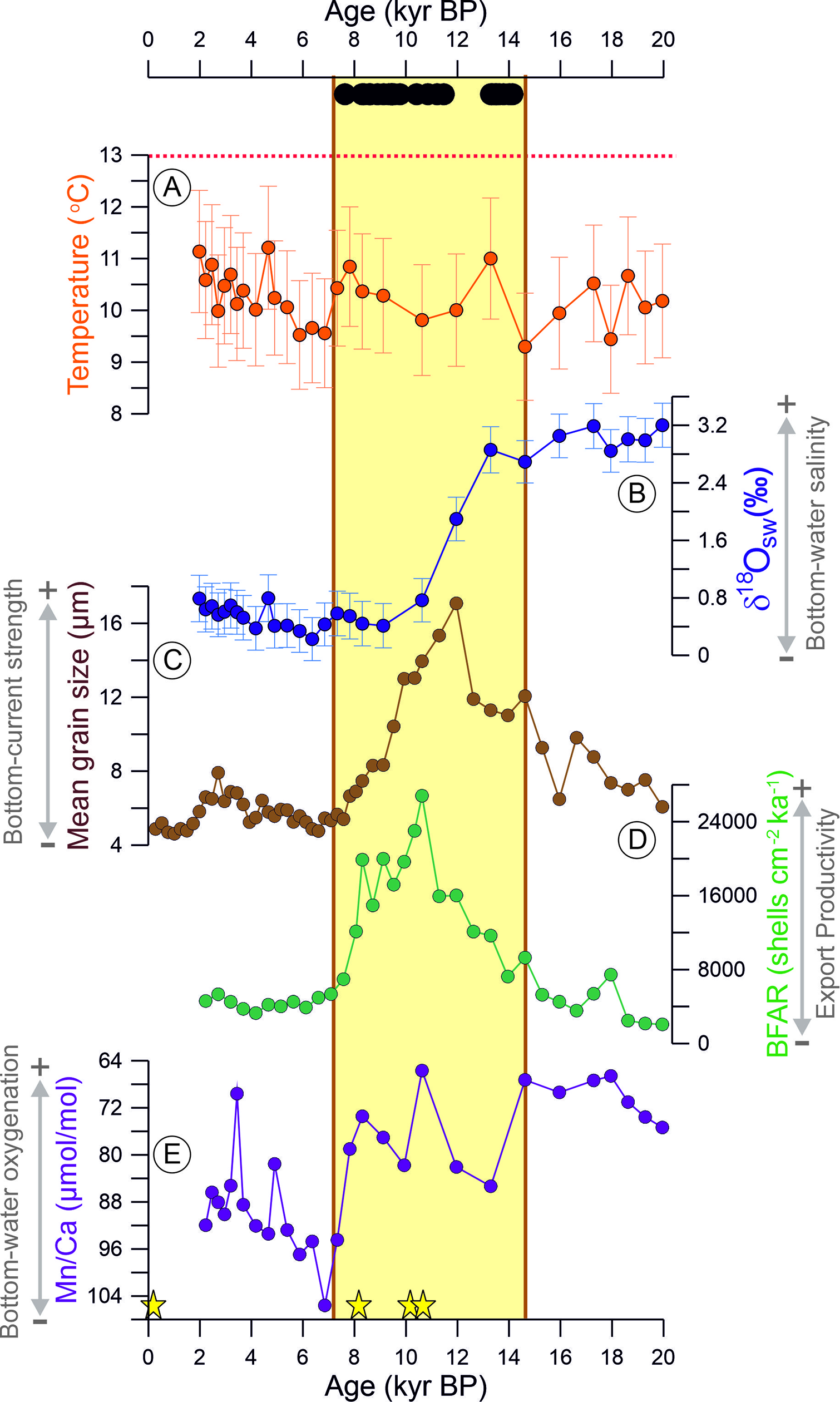

Supplement: S11 Fig — The occurrence of Lophelia pertusa in the region is indicated by black dots at the top (corresponding to AMS 14C and U/Th ages listed in S3 Table). The entire interval of coral growth is highlighted by the yellow vertical bar. The paleoceanographic proxies have been obtained from the off-mound core GeoB18131-1 [39]. (A) Bottom-water temperature is based on Mg/Ca ratios (for details, see S4 Table). (B) Bottom-water salinity is estimated from δ18OSW that was calculated from paired δ18O and Mg/Ca measurements (for details, see S4 Table). (C) The mean grain-size record as a proxy for the bottom current strength [39]. (D) The BFAR, based on foraminifera counting, is a proxy for organic matter flux to the seafloor. (E) Mn/Ca ratios measured on Cibicidoides mundulus are a proxy for bottom-water oxygenation (note the inverse axis). Horizontal dashed lines in (A) indicate local modern annual values of temperature at the core site in accordance to WOA2018 [4]. The vertical brown lines define the on- and/or offsets of coral growth. Calibrated AMS 14C ages for core GeoB18131-1 are shown as yellow stars at the bottom [39]. The underlying data for this figure can be found in https://doi.org/10.1594/PANGAEA.932775. BFAR, benthic foraminifera accumulation rate. (TIF) [file pbio.3001628.s011.tif]

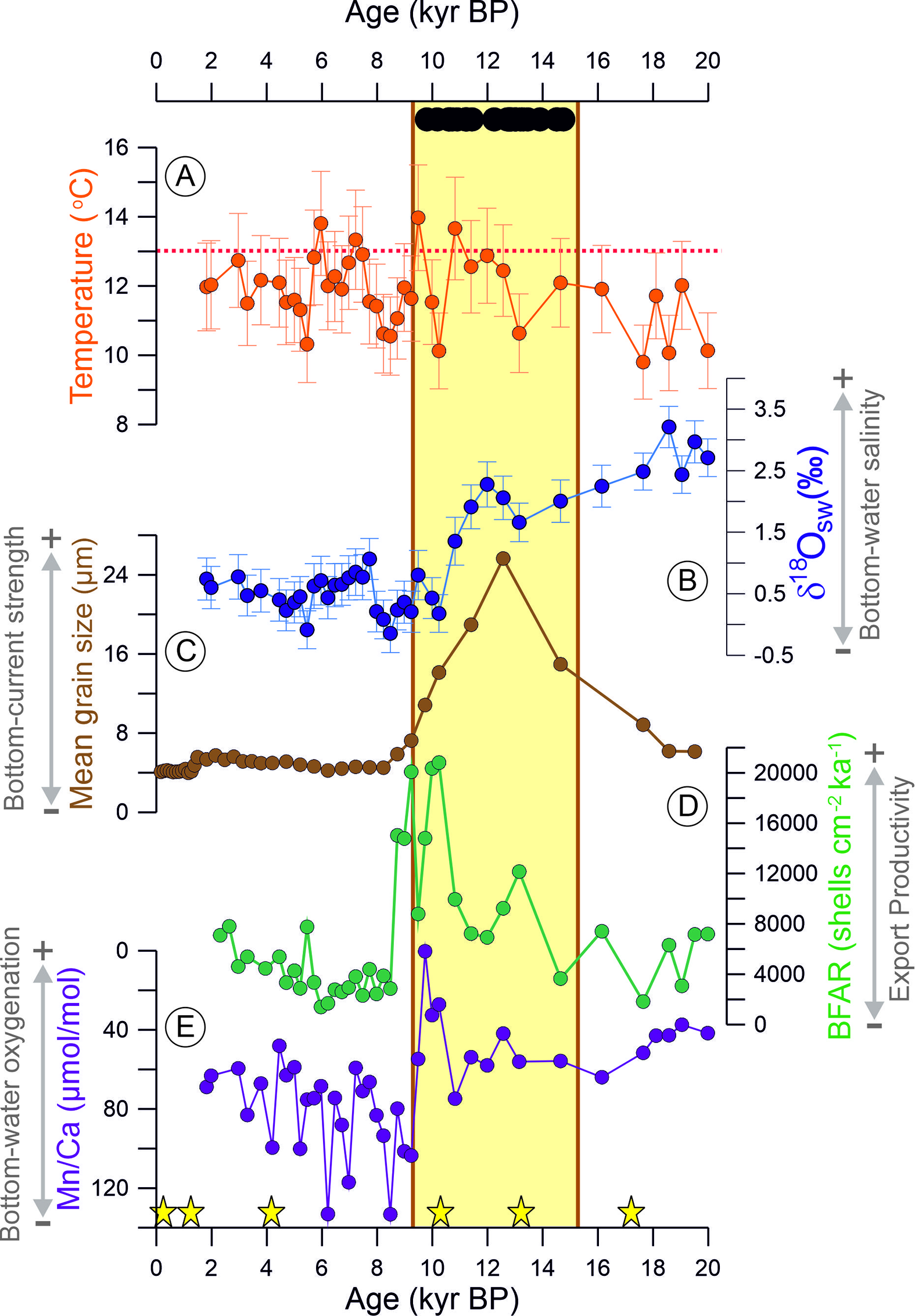

Supplement: S12 Fig — The occurrence of Lophelia pertusa in the region is indicated by black dots at the top (corresponding to AMS 14C and U/Th ages listed in S3 Table). The entire interval of coral growth is highlighted by the light yellow vertical bar. Paleoceanographic proxies have been obtained from off-mound core GeoB1373-1 [40]. (A) Bottom-water temperature is based on the Mg/Ca ratios (for details, see S4 Table). (B) Bottom-water salinity is estimated from δ18OSW that was calculated from paired δ18O and Mg/Ca measurements (for details, see S4 Table). (C) The mean grain-size record as a proxy for the bottom current strength [40]. (D) The BFAR, based on foraminifera counting, is a proxy for organic matter flux to the seafloor. (E) Mn/Ca ratios measured on Cibicidoides mundulus are a proxy for bottom-water oxygenation (note the inverse axis). Horizontal dashed lines in (A) indicate local modern annual values of temperature at the core site in accordance to WOA2018 [4]. The vertical brown lines define the on- and/or offsets of coral growth. Calibrated AMS 14C ages for core GeoB1373-1 are shown as yellow stars at the bottom [40]. The underlying data for this figure can be found in https://doi.org/10.1594/PANGAEA.932775. BFAR, benthic foraminifera accumulation rate. (TIF) [file pbio.3001628.s012.tif]
