# Supplementary material for: Major environmental drivers determining life and death of cold-water corals through time
Source: PLoS Biol. 2022 May 19;20(5):e3001628. doi: 10.1371/journal.pbio.3001628 (PMC9119455; doi:10.1371/journal.pbio.3001628)
Supplement: S1 Table — Further information are provided in the references listed to the right. CWC, cold-water coral. (DOCX) [file pbio.3001628.s013.docx]

|  | Cold-water coral sites | Sediment core | Latitude | Longitude | Water depth (m) | References |
| --- | --- | --- | --- | --- | --- | --- |
| NW-Atlantic | Gulf of Mexico (Campeche Bank) | GeoB 16320-2 | 23^°^83'N | 87^°^15'W | 626 | Matos et al. [1] |
| NE-Atlantic | Irish margin (Porcupine Seabight) | GeoB 6718-2 | 52^°^09'N | 12^°^45'W | 900 | Dorschel et al. [2] |
|  | Moroccan margin  (Gulf of Cadiz) | GeoB 9064-1 | 35^°^24'N | 06^°^50'W | 702 | Wienberg et al. [3] |
|  | Mauritanian margin | GeoB 14885-1 | 18^°^57'N | 16^°^52'W | 611 | This study |
| Mediterranean Sea | Alboran Sea  (West Melilla) | GeoB 18131-1 | 35^°^28'N | 03^°^09'W | 457 | Wang et al. [4] |
|  | Alboran Sea  (East Melilla) | GeoB 13731-1 | 35^°^24'N | 02^°^33'W | 362 | Fink et al. [5] |

**References:**

1. Matos L, Wienberg C, Titschack J, Schmiedl G, Frank N, Abrantes F, et al. Coral mound development at the Campeche cold-water coral province, southern Gulf of Mexico: Implications of Antarctic Intermediate Water increased influence during interglacials. Mar Geol. 2017;392: 53–65. doi:10.1016/j.margeo.2017.08.012

2. Dorschel B, Hebbeln D, Rüggeberg A, Dullo W, Freiwald A. Growth and erosion of a cold-water coral covered carbonate mound in the Northeast Atlantic during the Late Pleistocene and Holocene. Earth Planet Sci Lett. 2005;233: 33–44. doi:10.1016/j.epsl.2005.01.035

3. Wienberg C, Frank N, Mertens KN, Stuut J-B, Marchant M, Fietzke J, et al. Glacial cold-water coral growth in the Gulf of Cádiz: Implications of increased palaeo-productivity. Earth Planet Sci Lett. 2010;298: 405–416. doi:10.1016/j.epsl.2010.08.017

4. Wang H, Lo Iacono C, Wienberg C, Titschack J, Hebbeln D. Cold-water coral mounds in the southern Alboran Sea (western Mediterranean Sea): Internal waves as an important driver for mound formation since the last deglaciation. Mar Geol. 2019;412: 1–18. doi:10.1016/j.margeo.2019.02.007

5. Fink HG, Wienberg C, De Pol-Holz R, Wintersteller P, Hebbeln D. Cold-water coral growth in the Alboran Sea related to high productivity during the Late Pleistocene and Holocene. Mar Geol. 2013;339: 71–82. doi:10.1016/j.margeo.2013.04.009
