# Supplementary material for: Major environmental drivers determining life and death of cold-water corals through time
Source: PLoS Biol. 2022 May 19;20(5):e3001628. doi: 10.1371/journal.pbio.3001628 (PMC9119455; doi:10.1371/journal.pbio.3001628)
Supplement: S2 Table — (DOCX) [file pbio.3001628.s014.docx]

|  | Cold-water coral sites | Mg/Ca | δ^18^O | Grain Size | BFAR | Mn/Ca | Age model |
| --- | --- | --- | --- | --- | --- | --- | --- |
| NW-Atlantic | Gulf of Mexico (Campeche Bank) | This study | Matos et al. [1] | Matos et al. [1] | This study | This study | Matos et al. [1] |
| NE-Atlantic | Irish margin (Porcupine Seabight) | This study | Dorschel et al. [2] | Dorschel et al. [2] | This study | This study | This study |
|  | Moroccan margin  (Gulf of Cadiz) | This study | This study | Wienberg et al. [3] | This study | This study | Wienberg et al. [3] |
|  | Mauritanian margin | This study | This study | This study | This study | This study | This study |
| Mediterranean Sea | Alboran Sea  (West Melilla) | This study | Wang et al. [4] | Wang et al. [4] | Wang et al. [4] | This study | Wang et al. [4] |
|  | Alboran Sea  (East Melilla) | This study | Fink et al. [5] | Fink et al. [5] | This study | This study | Fink et al. [5] |

**References:**

1. Matos L, Wienberg C, Titschack J, Schmiedl G, Frank N, Abrantes F, et al. Coral mound development at the Campeche cold-water coral province, southern Gulf of Mexico: Implications of Antarctic Intermediate Water increased influence during interglacials. Mar Geol. 2017;392: 53–65. doi:10.1016/j.margeo.2017.08.012

2. Dorschel B, Hebbeln D, Rüggeberg A, Dullo W, Freiwald A. Growth and erosion of a cold-water coral covered carbonate mound in the Northeast Atlantic during the Late Pleistocene and Holocene. Earth Planet Sci Lett. 2005;233: 33–44. doi:10.1016/j.epsl.2005.01.035

3. Wienberg C, Frank N, Mertens KN, Stuut J-B, Marchant M, Fietzke J, et al. Glacial cold-water coral growth in the Gulf of Cádiz: Implications of increased palaeo-productivity. Earth Planet Sci Lett. 2010;298: 405–416. doi:10.1016/j.epsl.2010.08.017

4. Wang H, Lo Iacono C, Wienberg C, Titschack J, Hebbeln D. Cold-water coral mounds in the southern Alboran Sea (western Mediterranean Sea): Internal waves as an important driver for mound formation since the last deglaciation. Mar Geol. 2019;412: 1–18. doi:10.1016/j.margeo.2019.02.007

5. Fink HG, Wienberg C, De Pol-Holz R, Wintersteller P, Hebbeln D. Cold-water coral growth in the Alboran Sea related to high productivity during the Late Pleistocene and Holocene. Mar Geol. 2013;339: 71–82. doi:10.1016/j.margeo.2013.04.009
