# Supplementary material for: Major environmental drivers determining life and death of cold-water corals through time
Source: PLoS Biol. 2022 May 19;20(5):e3001628. doi: 10.1371/journal.pbio.3001628 (PMC9119455; doi:10.1371/journal.pbio.3001628)
Supplement: S3 Table — Coral fragments were collected from 6 coral mound provinces in the North Atlantic and the Mediterranean Sea. Only ages encompassing the last 20 kyr are listed and discussed for our study. AMS 14C coral ages obtained for the Irish margin and the Mediterranean Sea were recalibrated using the CALIB8.2 software [41]. For the calibration of the Irish coral ages, we have applied the MARINE20 calibration curve [42] with a local reservoir age correction of ΔR = −70 ± 50 years accounting for a marine reservoir age of R = 480 ± 120 years for the Holocene, which is based on paired AMS14C –U/Th dating published in Frank and colleagues [43,45]. For the western Mediterranean coral ages, we have applied the MARINE20 calibration curve with a local reservoir age correction of ΔR = −90 ± 80 years accounting for a deglacial to Holocene marine reservoir age of R = 370 ±40 years (according to Reimer and McCormac [46] and Siani and colleagues [47,48]). CWC, cold-water coral. (DOCX) [file pbio.3001628.s015.docx]

| Sample-ID | Sampling  depth (m) | Lab-Code | Dating  method | raw ^14^C age  (years) | σ | **cal. ^14^C age**  **(years BP)** | **2σ** | **U/Th-age**  **(years BP)** | **error** | original data source |
| --- | --- | --- | --- | --- | --- | --- | --- | --- | --- | --- |
| **Gulf of Mexico (Campeche Bank)** | | |  |  |  |  |  |  |  |  |
| GeoB16310-3 | 0.11 | LSCE-3370 | U/Th |  |  |  |  | **1,399** | **63** | Matos et al. [1] |
| GeoB16313-2 | 0.33 | IUPH-6795 | U/Th |  |  |  |  | **3,904** | **290** | Matos et al. [1] |
| GeoB16318-1 | 0.44 | IUPH-6788 | U/Th |  |  |  |  | **4,384** | **290** | Matos et al. [1] |
| GeoB16318-1 | 0.76 | IUPH-6789 | U/Th |  |  |  |  | **4,464** | **320** | Matos et al. [1] |
| GeoB16310-3 | 1.40 | LSCE-3416 | U/Th |  |  |  |  | **4,774** | **32** | Matos et al. [1] |
| GeoB16313-2 | 1.08 | IUPH-6797 | U/Th |  |  |  |  | **5,704** | **240** | Matos et al. [1] |
| GeoB16313-2 | 0.85 | IUPH-6796 | U/Th |  |  |  |  | **5,764** | **210** | Matos et al. [1] |
| GeoB16313-2 | 2.08 | IUPH-6798 | U/Th |  |  |  |  | **7,654** | **240** | Matos et al. [1] |
| GeoB16310-3 | 2.86 | LSCE-3418 | U/Th |  |  |  |  | **10,011** | **57** | Matos et al. [1] |
| **Irish margin (Porcupine Seabight)** | | |  |  |  |  |  |  |  |  |
| GeoB9214-1 | 0.20 | BETA 340730 | AMS^14^C | 1,430 | 40 | **880** | **180** |  |  | Wienberg et al.[2] |
| GeoB14535-2 | 0.00 | BETA 344869 | AMS^14^C | 2,060 | 30 | **1,540** | **190** |  |  | Wienberg et al.[2] |
| GeoB14535-2 | 0.00 | BETA 340739 | AMS^14^C | 2,170 | 30 | **1,660** | **190** |  |  | Wienberg et al.[2] |
| GeoB14539-1 | 0.00 | BETA 340740 | AMS^14^C | 3,370 | 30 | **3,140** | **210** |  |  | Wienberg et al.[2] |
| GeoB14539-1 | 0.00 | BETA 344874 | AMS^14^C | 3,880 | 30 | **3,760** | **210** |  |  | Wienberg et al.[2] |
| GeoB14531-1 | 2.07 | BETA 340736 | AMS^14^C | 5,920 | 30 | **6,220** | **210** |  |  | Wienberg et al.[2] |
| GeoB14530-1 | 1.80 | BETA 340735 | AMS^14^C | 6,060 | 30 | **6,360** | **180** |  |  | Wienberg et al.[2] |
| GeoB14539-1 | 0.00 | BETA 340742 | AMS^14^C | 6,980 | 30 | **7,360** | **180** |  |  | Wienberg et al.[2] |
| GeoB14546-1 | 0.58 | BETA 340744 | AMS^14^C | 7,250 | 30 | **7,600** | **170** |  |  | Wienberg et al.[2] |
| GeoB14550-1 | 0.06 | BETA 340745 | AMS^14^C | 7,480 | 30 | **7,830** | **170** |  |  | Wienberg et al.[2] |
| MD01-2463G | 0.00 | GIF | U/Th |  |  |  |  | **202** | **33** | Frank et al. [3] |
| GeoB14531-1 | 0.13 | GIF-3054 | U/Th |  |  |  |  | **352** | **15** | Wienberg et al.[2] |
| 2419 | 0.00 | IUPH | U/Th |  |  |  |  | **405** | **60** | Schröder-Ritzrau et al. [51] |
| GeoB14530-1 | 0.19 | GIF-6065 | U/Th |  |  |  |  | **953** | **46** | Wienberg et al.[2] |
| 2420 | 0.00 | IUPH | U/Th |  |  |  |  | **995** | **190** | Schröder-Ritzrau et al.[4] |
| IODP 1317 | 0.10 | 900-09/650-10 | U/Th |  |  |  |  | **1,176** | **30** | Raddatz et al. [5] |
| GeoB14532-2 | 0.67 | GIF-3069 | U/Th |  |  |  |  | **2,924** | **31** | Wienberg et al. (2020) [2] |
| MD01-2451G | 0.06 | GIF | U/Th |  |  |  |  | **3,174** | **106** | Frank et al. [3] |
| GeoB14531-1 | 0.98 | GIF-3055 | U/Th |  |  |  |  | **4,227** | **27** | Wienberg et al.[2] |
| MD01-2459G | 0.00 | GIF | U/Th |  |  |  |  | **4,309** | **95** | Frank et al. [3] |
| GeoB14532-2 | 0.20 | GIF-3067 | U/Th |  |  |  |  | **4,838** | **89** | Wienberg et al.[2] |
| GeoB14532-2 | 0.43 | GIF-3068 | U/Th |  |  |  |  | **5,571** | **43** | Wienberg et al.[2] |
| GeoB9213-1 | 0.51 | GIF-3058 | U/Th |  |  |  |  | **5,628** | **55** | Wienberg et al.[2] |
| MD01-2451G | 0.27 | GIF | U/Th |  |  |  |  | **6,051** | **153** | Frank et al. [3] |
| MD01-2451G | 0.31 | GIF | U/Th |  |  |  |  | **6,461** | **140** | Frank et al. [3] |
| GeoB9213-1 | 1.04 | GIF-3059 | U/Th |  |  |  |  | **6,577** | **45** | Wienberg et al.[2] |
| MD01-2459G | 0.50 | GIF | U/Th |  |  |  |  | **7,481** | **180** | Frank et al. [3] |
| MD01-2459G | 1.50 | GIF | U/Th |  |  |  |  | **8,461** | **100** | Frank et al. [3] |
| MD01-2459G | 1.12 | GIF | U/Th |  |  |  |  | **8,706** | **190** | Frank et al. [3] |
| GeoB14531-1 | 4.34 | GIF-3057 | U/Th |  |  |  |  | **8,859** | **59** | Wienberg et al.[2] |
| MD01-2459G | 2.05 | GIF | U/Th |  |  |  |  | **8,871** | **170** | Frank et al. [3] |
| GeoB9213-1 | 1.50 | GEOMAR | U/Th |  |  |  |  | **9,112** | **150** | Eisele et al. [6] |
| GeoB9213-1 | 2.50 | GIF-3060 | U/Th |  |  |  |  | **9,195** | **71** | Wienberg et al.[2] |
| MD01-2459G | 3.00 | GIF | U/Th |  |  |  |  | **9,231** | **210** | Frank et al. [3] |
| GeoB14531-1 | 3.20 | GIF-3056 | U/Th |  |  |  |  | **9,319** | **44** | Wienberg et al.[2] |
| MD01-2463G | 1.79 | GIF | U/Th |  |  |  |  | **9,641** | **350** | Frank et al. [3] |
| MD01-2459G | 4.50 | GIF | U/Th |  |  |  |  | **9,683** | **74** | Frank et al. [3] |
| MD01-2463G | 1.41 | GIF | U/Th |  |  |  |  | **9,751** | **220** | Frank et al.[3] |
| GeoB14530-1 | 3.47 | GIF-3066 | U/Th |  |  |  |  | **11,290** | **92** | Wienberg et al.[2] |
| **Moroccan margin (Gulf of Cádiz)** | | |  |  |  |  |  |  |  |  |
| MD08-3212 | 0.08 | GIF | U/Th |  |  |  |  | **11,022** | **230** | Frank et al. [7] |
| B09 1406 | 0.15 | GIF | U/Th |  |  |  |  | **11,972** | **350** | Frank et al. [7] |
| B09 1406 | 0.10 | GIF | U/Th |  |  |  |  | **12,372** | **810** | Frank et al. [7] |
| B09 1408 | 0.38 | GIF | U/Th |  |  |  |  | **14,082** | **590** | Frank et al. [7] |
| MD08-3212 | 0.21 | GIF | U/Th |  |  |  |  | **14,272** | **690** | Frank et al. [7] |
| B09 1405 | 0.30 | GIF | U/Th |  |  |  |  | **14,282** | **510** | Frank et al. [7] |
| MD08-3231 | 0.03 | GIF | U/Th |  |  |  |  | **14,724** | **400** | Frank et al. [7] |
| B09 1408 | 0.00 | GIF | U/Th |  |  |  |  | **15,042** | **380** | Frank et al. [7] |
| B09 1405 | 0.15 | GIF | U/Th |  |  |  |  | **15,142** | **380** | Frank et al. [7] |
| M2005-31 | 0.00 | GIF | U/Th |  |  |  |  | **15,152** | **230** | Frank et al. [7] |
| M2004-02 | 0.85 | GIF-1632 | U/Th |  |  |  |  | **19,250** | **560** | Wienberg et al. [8] |
| B09 1405 | 0.10 | GIF | U/Th |  |  |  |  | **19,582** | **500** | Frank et al. [7] |
| M2004-02 | 1.05 | GIF-1633 | U/Th |  |  |  |  | **19,743** | **540** | Wienberg et al. [8] |
| **Mauritanian margin** | |  |  | U/Th |  |  |  |  |  |  |
| GeoB 14799-2 | 0.51 | GIF-2786 | U/Th |  |  |  |  | **11,313** | **28** | Wienberg et al. [9] |
| GeoB 14799-2 | 1.16 | GIF-2788 | U/Th |  |  |  |  | **11,346** | **43** | Wienberg et al. [9] |
| GeoB 14799-2 | 0.95 | GIF-2787 | U/Th |  |  |  |  | **11,352** | **36** | Wienberg et al. [9] |
| GeoB 14799-2 | 0.04 | GIF-2785 | U/Th |  |  |  |  | **11,381** | **41** | Wienberg et al. [9] |
| GeoB 14878-1 | 0.00 | GIF-2450 | U/Th |  |  |  |  | **11,447** | **43** | Wienberg et al. [9] |
| GeoB 14904-2 | 0.01 | GIF-2793 | U/Th |  |  |  |  | **12,463** | **50** | Wienberg et al. [9] |
| GeoB 14873-3 | 0.00 | GIF-2452 | U/Th |  |  |  |  | **12,471** | **44** | Wienberg et al. [9] |
| GeoB 14897-1 | 0.00 | GIF-2461 | U/Th |  |  |  |  | **12,776** | **32** | Wienberg et al. [9] |
| GeoB 14905-2 | 0.01 | IUP-8230 | U/Th |  |  |  |  | **13,063** | **63** | Wienberg et al. [9] |
| GeoB 14877-1 | 0.00 | GIF-2449 | U/Th |  |  |  |  | **13,118** | **63** | Wienberg et al. [9] |
| GeoB 14905-2 | 1.99 | IUP-8231 | U/Th |  |  |  |  | **13,600** | **71** | Wienberg et al. [9] |
| GeoB 14904-1 | 0.00 | GIF-2464 | U/Th |  |  |  |  | **13,712** | **75** | Wienberg et al. [9] |
| GeoB 14904-2 | 0.67 | GIF-2795 | U/Th |  |  |  |  | **13,912** | **71** | Wienberg et al. [9] |
| GeoB 14904-2 | 1.32 | GIF-2796 | U/Th |  |  |  |  | **13,951** | **66** | Wienberg et al. [9] |
| GeoB 14905-2 | 2.81 | IUP-8232 | U/Th |  |  |  |  | **14,111** | **87** | Wienberg et al. [9] |
| GeoB 11569-2 | 0.13 | GIF-1187 | U/Th |  |  |  |  | **14,182** | **220** | Eisele et al. [10] |
| GeoB 14905-2 | 4.29 | IUP-8233 | U/Th |  |  |  |  | **14,239** | **58** | Wienberg et al. [9] |
| GeoB 14904-2 | 2.93 | GIF-2799 | U/Th |  |  |  |  | **18,592** | **85** | Wienberg et al. [9] |
| GeoB 14904-2 | 2.16 | GIF-2797 | U/Th |  |  |  |  | **18,724** | **75** | Wienberg et al. [9] |
| GeoB 14904-2 | 3.56 | GIF-2800 | U/Th |  |  |  |  | **18,758** | **103** | Wienberg et al. [9] |
| GeoB 14904-2 | 2.45 | GIF-2798 | U/Th |  |  |  |  | **19,235** | **95** | Wienberg et al. [9] |
| GeoB 14884-1 | 0.19 | IUP-8052 | U/Th |  |  |  |  | **19,674** | **69** | Wienberg et al. [9] |
| GeoB 14904-2 | 4.35 | GIF-2801 | U/Th |  |  |  |  | **19,732** | **95** | Wienberg et al. [9] |
| **Alboran Sea (West Melilla)** | |  |  |  |  |  |  |  |  |  |
| MD13-3451G | 1.97 | Poz-62333 | AMS^14^C | 8,900 | 40 | **9,510** | **250** |  |  | Wang et al. [11] |
| MD13-3452G | 2.67 | Poz-62339 | AMS^14^C | 9,900 | 40 | **10,870** | **290** |  |  | Wang et al. [11] |
| MD13-3451G | 3.82 | Poz-62334 | AMS^14^C | 10,390 | 50 | **11,530** | **310** |  |  | Wang et al. [11] |
| MD13-3452G | 4.06 | Poz-62341 | AMS^14^C | 11,830 | 70 | **13,240** | **250** |  |  | Wang et al. [11] |
| MD13-3452G | 4.67 | Poz-62342 | AMS^14^C | 12,170 | 50 | **13,610** | **280** |  |  | Wang et al. [11] |
| MD13-3451G | 4.88 | Poz-62335 | AMS^14^C | 12,320 | 50 | **13,880** | **300** |  |  | Wang et al. [11] |
| MD13-3452G | 5.50 | Poz-62343 | AMS^14^C | 12,540 | 50 | **14,100** | **330** |  |  | Wang et al. [11] |
| GeoB18127-1 | 0.52 | IUP-7736 | U/Th |  |  |  |  | **7,567** | **199** | Wang et al. [11] |
| GeoB18127-1 | 0.79 | IUP-7738 | U/Th |  |  |  |  | **8,236** | **139** | Wang et al. [11] |
| GeoB18127-1 | 0.90 | IUP-7739 | U/Th |  |  |  |  | **8,305** | **123** | Wang et al. [11] |
| GeoB18127-1 | 1.59 | IUP-7740 | U/Th |  |  |  |  | **8,512** | **174** | Wang et al. [11] |
| GeoB18127-1 | 1.89 | IUP-7741 | U/Th |  |  |  |  | **8,554** | **122** | Wang et al. [11] |
| GeoB18130-1 | 0.71 | IUP-7754 | U/Th |  |  |  |  | **8,829** | **48** | Wang et al. [11] |
| GeoB18130-1 | 1.18 | IUP-7756 | U/Th |  |  |  |  | **9,069** | **53** | Wang et al. [11] |
| GeoB18127-1 | 2.52 | IUP-7742 | U/Th |  |  |  |  | **9,280** | **144** | Wang et al. [11] |
| GeoB18130-1 | 1.28 | IUP-7757 | U/Th |  |  |  |  | **9,375** | **52** | Wang et al. [11] |
| GeoB18130-1 | 1.38 | IUP-7758 | U/Th |  |  |  |  | **9,383** | **50** | Wang et al. [11] |
| GeoB18130-1 | 1.47 | IUP-7759 | U/Th |  |  |  |  | **9,397** | **52** | Wang et al. [11] |
| GeoB18127-1 | 2.74 | IUP-7743 | U/Th |  |  |  |  | **9,705** | **84** | Wang et al. [11] |
| GeoB18127-1 | 3.37 | IUP-7744 | U/Th |  |  |  |  | **10,350** | **92** | Wang et al. [11] |
| GeoB18127-1 | 3.73 | IUP-7745 | U/Th |  |  |  |  | **11,146** | **69** | Wang et al. [11] |
| GeoB18127-1 | 4.04 | IUP-7746 | U/Th |  |  |  |  | **13,300** | **97** | Wang et al. [11] |
| GeoB18127-1 | 4.60 | IUP-7747 | U/Th |  |  |  |  | **13,402** | **103** | Wang et al. [11] |
| GeoB18127-1 | 4.78 | IUP-7748 | U/Th |  |  |  |  | **13,456** | **87** | Wang et al. [11] |
| GeoB18127-1 | 4.91 | IUP-7749 | U/Th |  |  |  |  | **14,075** | **114** | Wang et al. [11] |
| **Alboran Sea (East Melilla)** | |  |  |  |  |  |  |  |  |  |
| GeoB 13729-1 | 0.03 | UCIAMS-73570 | AMS^14^C | 9,085 | 30 | **9,740** | **250** |  |  | Fink et al. [12] |
| GeoB 13729-1 | 0.49 | UCIAMS-73571 | AMS^14^C | 9,330 | 25 | **10,070** | **310** |  |  | Fink et al. [12] |
| GeoB 13730-1 | 1.02 | UCIAMS-73576 | AMS^14^C | 9,700 | 25 | **10,570** | **320** |  |  | Fink et al. [12] |
| GeoB 13729-1 | 1.40 | UCIAMS-73572 | AMS^14^C | 9,705 | 25 | **10,580** | **330** |  |  | Fink et al. [12] |
| GeoB 13730-1 | 1.94 | UCIAMS-73577 | AMS^14^C | 9,810 | 25 | **10,740** | **280** |  |  | Fink et al. [12] |
| GeoB 13729-1 | 3.15 | UCIAMS-73573 | AMS^14^C | 9,935 | 30 | **10,910** | **280** |  |  | Fink et al. [12] |
| GeoB 13729-1 | 3.75 | UCIAMS-73574 | AMS^14^C | 10,225 | 30 | **11,310** | **240** |  |  | Fink et al. [12] |
| GeoB 13730-1 | 2.98 | UCIAMS-73578 | AMS^14^C | 10,230 | 25 | **11,310** | **240** |  |  | Fink et al. [12] |
| GeoB 13728-2 | 0.35 | UCIAMS-96720 | AMS^14^C | 10,280 | 20 | **11,380** | **240** |  |  | Fink et al. [12] |
| GeoB 13730-1 | 3.43 | UCIAMS-96723 | AMS^14^C | 10,390 | 20 | **11,530** | **300** |  |  | Fink et al. [12] |
| TTR-401G | 2.60 | ETH-55623 | AMS^14^C | 10,770 | 39 | **12,140** | **320** |  |  | Stalder et al. [13] |
| TTR-401G | 4.20 | ETH-55625 | AMS^14^C | 11,231 | 39 | **12,670** | **210** |  |  | Stalder et al. [13] |
| GeoB 13728-2 | 1.17 | UCIAMS-96721 | AMS^14^C | 11,310 | 25 | **12,740** | **190** |  |  | Fink et al. [12] |
| GeoB 13730-1 | 3.95 | UCIAMS-96724 | AMS^14^C | 11,335 | 20 | **12,770** | **180** |  |  | Fink et al. [12] |
| GeoB 13728-2 | 1.32 | UCIAMS-96722 | AMS^14^C | 11,385 | 25 | **12,810** | **180** |  |  | Fink et al. [12] |
| GeoB 13728-2 | 1.91 | UCIAMS-73568 | AMS^14^C | 11,415 | 30 | **12,840** | **180** |  |  | Fink et al. [12] |
| GeoB 13737#2_A | 0.00 | UCIAMS-67193 | AMS^14^C | 11,515 | 30 | **12,930** | **200** |  |  | Stalder et al. [13] |
| TTR-401G | 5.60 | ETH-55627 | AMS^14^C | 11,553 | 40 | **12,960** | **220** |  |  | Stalder et al. [13] |
| GeoB 13728-2 | 2.95 | UCIAMS-73569 | AMS^14^C | 11,700 | 30 | **13,110** | **230** |  |  | Fink et al. [12] |
| GeoB 13730-1 | 4.27 | UCIAMS-73579 | AMS^14^C | 11,825 | 30 | **13,240** | **200** |  |  | Fink et al. [12] |
| GeoB 13737#1_A | 0.00 | UCIAMS-67192 | AMS^14^C | 12,025 | 35 | **13,440** | **230** |  |  | Stalder et al. [13] |
| MD13-3455G | 0.35 | IUP-8489 | U/Th |  |  |  |  | **13,725** | **49** | Fentimen et al. [14] |
| GP21 | 0.15 | n/a | U/Th |  |  |  |  | **13,900** | **60** | Dubois-Dauphin et al. [15] |
| MD13-3455G | 2.80 | IUP-8490 | U/Th |  |  |  |  | **14,501** | **54** | Fentimen et al. [14] |
| MD13-3455G | 3.28 | IUP-8491 | U/Th |  |  |  |  | **14,519** | **52** | Fentimen et al. [14] |
| MD13-3455G | 4.45 | IUP-8492 | U/Th |  |  |  |  | **14,747** | **52** | Fentimen et al. [14] |

**References:**

1. Matos L, Wienberg C, Titschack J, Schmiedl G, Frank N, Abrantes F, et al. Coral mound development at the Campeche cold-water coral province, southern Gulf of Mexico: Implications of Antarctic Intermediate Water increased influence during interglacials. Mar Geol. 2017;392: 53–65. doi:10.1016/j.margeo.2017.08.012

2. Wienberg C, Titschack J, Frank N, De Pol-Holz R, Fietzke J, Eisele M, et al. Deglacial upslope shift of NE Atlantic intermediate waters controlled slope erosion and cold-water coral mound formation (Porcupine Seabight, Irish margin). Quat Sci Rev. 2020;237: 106310. doi:10.1016/j.quascirev.2020.106310

3. Frank N, Ricard E, Lutringer-Paquet A, van der Land C, Colin C, Blamart D, et al. The Holocene occurrence of cold water corals in the NE Atlantic: Implications for coral carbonate mound evolution. Mar Geol. 2009;266: 129–142. doi:10.1016/j.margeo.2009.08.007

4. Schröder-Ritzrau A, Freiwald A, Mangini A. U/Th-dating of deep-water corals from the eastern North Atlantic and the western Mediterranean Sea. Cold-Water Corals and Ecosystems. Berlin/Heidelberg: Springer-Verlag; 2005. pp. 157–172. doi:10.1007/3-540-27673-4_8

5. Raddatz J, Rüggeberg A, Liebetrau V, Foubert A, Hathorne EC, Fietzke J, et al. Environmental boundary conditions of cold-water coral mound growth over the last 3 million years in the Porcupine Seabight, Northeast Atlantic. Deep Sea Res Part II Top Stud Oceanogr. 2014;99: 227–236. doi:10.1016/j.dsr2.2013.06.009

6. Eisele M, Hebbeln D, Wienberg C. Growth history of a cold-water coral covered carbonate mound — Galway Mound, Porcupine Seabight, NE-Atlantic. Mar Geol. 2008;253: 160–169. doi:10.1016/j.margeo.2008.05.006

7. Frank N, Freiwald A, López Correa M, Wienberg C, Eisele M, Hebbeln D, et al. Northeastern Atlantic cold-water coral reefs and climate. Geology. 2011;39: 743–746. doi:10.1130/G31825.1

8. Wienberg C, Frank N, Mertens KN, Stuut J-B, Marchant M, Fietzke J, et al. Glacial cold-water coral growth in the Gulf of Cádiz: Implications of increased palaeo-productivity. Earth Planet Sci Lett. 2010;298: 405–416. doi:10.1016/j.epsl.2010.08.017

9. Wienberg C, Titschack J, Freiwald A, Frank N, Lundälv T, Taviani M, et al. The giant Mauritanian cold-water coral mound province: Oxygen control on coral mound formation. Quat Sci Rev. 2018;185: 135–152. doi:10.1016/j.quascirev.2018.02.012

10. Eisele M, Frank N, Wienberg C, Hebbeln D, López Correa M, Douville E, et al. Productivity controlled cold-water coral growth periods during the last glacial off Mauritania. Mar Geol. 2011;280: 143–149. doi:10.1016/j.margeo.2010.12.007

11. Wang H, Lo Iacono C, Wienberg C, Titschack J, Hebbeln D. Cold-water coral mounds in the southern Alboran Sea (western Mediterranean Sea): Internal waves as an important driver for mound formation since the last deglaciation. Mar Geol. 2019;412: 1–18. doi:10.1016/j.margeo.2019.02.007

12. Fink HG, Wienberg C, De Pol-Holz R, Wintersteller P, Hebbeln D. Cold-water coral growth in the Alboran Sea related to high productivity during the Late Pleistocene and Holocene. Mar Geol. 2013;339: 71–82. doi:10.1016/j.margeo.2013.04.009

13. Stalder C, Vertino A, Rosso A, Rüggeberg A, Pirkenseer C, Spangenberg JE, et al. Microfossils, a Key to Unravel Cold-Water Carbonate Mound Evolution through Time: Evidence from the Eastern Alboran Sea. Abramovich S, editor. PLoS One. 2015;10: e0140223. doi:10.1371/journal.pone.0140223

14. Fentimen R, Feenstra E, Rüggeberg A, Vennemann T, Hajdas I, Adatte T, et al. Cold-Water Coral Mound Archive Provides Unique Insights Into Intermediate Water Mass Dynamics in the Alboran Sea During the Last Deglaciation. Front Mar Sci. 2020;7: 1–25. doi:10.3389/fmars.2020.00354

15. Dubois-Dauphin Q, Montagna P, Siani G, Douville E, Wienberg C, Hebbeln D, et al. Hydrological variations of the intermediate water masses of the western Mediterranean Sea during the past 20 ka inferred from neodymium isotopic composition in foraminifera and cold-water corals. Clim Past. 2017;13: 17–37. doi:10.5194/cp-13-17-2017
