# Supplementary material for: Major environmental drivers determining life and death of cold-water corals through time
Source: PLoS Biol. 2022 May 19;20(5):e3001628. doi: 10.1371/journal.pbio.3001628 (PMC9119455; doi:10.1371/journal.pbio.3001628)
Supplement: S4 Table — The underlying data for this figure can be found in https://doi.org/10.1594/PANGAEA.932775. (DOCX) [file pbio.3001628.s016.docx]

| Cores | Lab. No. | Core depth (cm) | Analyzed material | Radiocarbon age  ± 1σ error (kyr) | Calibrated age range  ± 2σ error (kyr BP) | Calibrated age  (kyr BP) | Observation |
| --- | --- | --- | --- | --- | --- | --- | --- |
| GeoB14885-1 | YAUT-029932 | 3 | Planktonic foraminifera | 2.60 ± 0.54 | 1.19 – 3.81 | 2.41 |  |
| GeoB14885-1 | YAUT-029931 | 88 | Planktonic foraminifera | 14.35 ± 0.86 | 14.29 – 18.82 | 16.67 |  |
| GeoB14885-1 | YAUT-029929 | 103 | Planktonic foraminifera | 13.52 ± 0.68 | 13.61 – 17.46 | 15.54 | Inversion |
| GeoB14885-1 | YAUT-029933 | 128 | Planktonic foraminifera | 17.22 ± 1.09 | 18.08 – 22.96 | 20.31 |  |
| GeoB6718-2 | AWI 2005.1.1 | 3 | Planktonic foraminifera | 1.34 ± 0.52 | 0 – 1.82 | 0.87 |  |
| GeoB6718-2 | AWI 2006.1.1 | 53 | Planktonic foraminifera | 5.66 ± 0.54 | 4.65 – 7.25 | 5.93 |  |
| GeoB6718-2 | AWI 2007.1.1 | 108 | Planktonic foraminifera | 6.50 ± 0.54 | 5.75 – 7.93 | 6.85 | Outlier |

* Mean of the 2σ calibrated age range.
