# Supplementary material for: Major environmental drivers determining life and death of cold-water corals through time
Source: PLoS Biol. 2022 May 19;20(5):e3001628. doi: 10.1371/journal.pbio.3001628 (PMC9119455; doi:10.1371/journal.pbio.3001628)
Supplement: S5 Table — The number in the left column refers to the regions presented in Fig 1 to which the respective equations have been applied. References: Shackleton [60]–(SK74), Cacho and colleagues [17]–(CA06), Bryan and Marchitto [13]–(BM08), Huang and colleagues [15]–(HU12), and Marchitto and colleagues [19]–(MA14). BWS, bottom-water paleosalinity; BWT, bottom-water paleotemperature. (DOCX) [file pbio.3001628.s017.docx]

| Region | Applied to species | Shell Size | Paleotemperature equation | Species calibration | Ref. | δ^18^O_SW_ equation | Species calibration | Ref. |
| --- | --- | --- | --- | --- | --- | --- | --- | --- |
| 1 | *Planulina ariminensis* | ≥250 | BWT=(LN(Mg/Ca/1))/0.08 | *P. ariminensis* | BM08 | δ^18^O_SW_=0.224*BWT-3.5+δ^18^O_C_ | *Cibicidoides* spp | MA14 |
| 2 | *Cibicides* spp | ≥250 | BWT=(Mg/Ca+0.06)/0.19 | *P. ariminensis* | HU12 | δ^18^O_SW_=δ^18^O_C_ +(BWT-16.9/4) +0.22 | *Cibicides* spp | HU12 |
| 3 | *Uvigerina* spp | ≥150 | BWT= (Mg/Ca+0.77)/0.08 | *Uvigerina* spp | BM08 | δ^18^O_SW_=0.231*BWT-4.03+δ^18^O_C_ | *Uvigerina* spp | MA14 |
| 4 | *Planulina ariminensis* | ≥205 | BWT=(LN(Mg/Ca/0.2))/0.184 | *P. ariminensis* | HU12 | δ^18^O_SW_=δ^18^O_C_ +(BWT-16.9/4) +0.22 | *Cibicides* spp | HU12 |
| 5 | *Cibicides mundulus* | ≥250 | BWT=(LN(Mg/Ca/0.61))/0.11 | *Cibicidoides* spp | CA06 | δ^18^O_SW_=δ^18^O_C_ +(BWT-16.9/4) | *Uvigerina* spp | SK74 |
| 6 | *Cibicides mundulus* | ≥250 | BWT=(LN(Mg/Ca/0.61))/0.11 | *Cibicidoides* spp | CA06 | δ^18^O_SW_=δ^18^O_C_ +(BWT-16.9/4) | *Uvigerina* spp | SK74 |
