# Supplementary material for: Major environmental drivers determining life and death of cold-water corals through time
Source: PLoS Biol. 2022 May 19;20(5):e3001628. doi: 10.1371/journal.pbio.3001628 (PMC9119455; doi:10.1371/journal.pbio.3001628)
Supplement: S6 Table — Values marked with a superscripted asterisk denote significance at a Dunn–Sidak corrected significance level of 0.05, and superscripted plus denotes significance at a Dunn–Sidak corrected significance level of 0.1. The underlying data for this figure can be found in https://doi.org/10.1594/PANGAEA.932775. NLR, nominal logistic regression. (DOCX) [file pbio.3001628.s018.docx]

|  | Cold-water coral sites | Temperature | δ^18^O  (salinity) | Hydrodynamic | Export Productivity | Oxygenation |
| --- | --- | --- | --- | --- | --- | --- |
| North Atlantic Ocean | Gulf of Mexico (Campeche Bank) | 0.399724 | 0.532728 | 0.350986 | 0.375887 | <0.0001^*^ |
|  | Irish margin (Porcupine Seabight) | 0.779808 | 0.031719 | 0.005626^*^ | 0.006868^*^ | 0.889478 |
|  | Moroccan margin  (Gulf of Cadiz) | 0.125086 | 0.088184 | 0.3507 | 0.142914 | 0.130796 |
|  | Mauritanian margin | 0.31103 | 0.336025 | 0.158029 | 0.078984 | 0.763353 |
| Mediterranean Sea | Alboran Sea  (West Melilla) | 0.707863 | 0.570355 | 0.00086^*^ | 0.077507 | 0.246704 |
|  | Alboran Sea  (East Melilla) | 0.122149 | 0.76546 | 0.002531^*^ | 0.010487^+^ | 0.228583 |
