# Supplementary material for: Major environmental drivers determining life and death of cold-water corals through time
Source: PLoS Biol. 2022 May 19;20(5):e3001628. doi: 10.1371/journal.pbio.3001628 (PMC9119455; doi:10.1371/journal.pbio.3001628)
Supplement: S1 Text — (DOCX) [file pbio.3001628.s019.docx]

Major environmental drivers determining life and death of cold-water corals through time

Rodrigo da Costa Portilho-Ramos^1^*, Jürgen Titschack^1,2^, Claudia Wienberg^1^, Michael Georg Siccha Rojas^1^, Yusuke Yokoyama^3^ and Dierk Hebbeln^1^

^1^MARUM – Center for Marine Environmental Sciences, University of Bremen, Bremen, Germany.

^2^Senckenberg am Meer, Marine Research Department, Wilhelmshaven, Germany.

^3^Analytical Center for Environmental Science – Atmosphere and Ocean Research Institute, University of Tokyo, Tokyo, Japan.

Supplementary Text

Material and Methods

In order to provide a comprehensive data set for all regions, we combined already published records with newly established data. Details on the sediment cores used and an overview about already existing and newly established data sets are provided in the S1 Table and S2 Table. To obtain continuous paleoenvironmental records, we used so-called off-mound cores, collected close to CWC mounds. Further, we compared the temporal occurrence of *Lophelia pertusa* (based on a compilation of previously published AMS ^14^C and U/Th dates; S3 Table), at these coral mounds with the ambient paleoenvironmental conditions obtained from the corresponding off-mound cores following the approach outlined by Hebbeln et al. [1]. All off-mound cores were collected at intermediate water depths between 360–900 m with annual mean bottom water temperatures of 5°–14°C, salinities of 33–39 psu, and oxygen contents of 1−7 ml l^-1^ from oligotrophic (Gulf of Mexico) to eutrophic (Mauritanian margin) regions (ranges of temperature, salinity and oxygen concentration are extracted from WOA18 [2] at the study sites with 1° resolution [3–5]. Detailed information of these marine sediment cores is listed in S1 Table.

***Age model***

Most off-mound sediment cores are presented in their originally published chronologies based on linear interpolation of AMS ^14^C ages supported by δ^18^O data (see references in S2 Table). Here, we provide seven new AMS ^14^C dates: four for the off-mound core GeoB14885-1 from the Mauritanian margin and three for the off-mound core GeoB6718-2 from the Irish margin (Porcupine Seabight) (S4 Table). For all measurements, we used mixed planktonic foraminifera. The AMS ^14^C datings for core GeoB14885-1 were performed at the University of Tokyo, Japan, while those from core GeoB6718-2 were performed on the Accelerator Mass Spectrometer "MICADAS" system at the Alfred Wegener Institute (AWI) facilities. The raw ages were calibrated into kiloyear before present (kyr BP) using the software PaleoDataView version 0.8.3.5 [6], with the Incal20 radiocarbon calibration curve [7] and variable simulated reservoir ages from transient modeling experiments described in Butzin et al. [8]. These new AMS^14^C as well as the calibrated ages can be found in S4 Table. Over the last 20kyr BP, the differences among our calibration and the originally published chronologies (Marine09 and Marine13) is less than 400 years. As we are not interested in the exact timing of presence and absence of CWC and are not comparing the timing of these events between sites, this age uncertainty does not affect our study.

In the Mauritanian core GeoB14885-1, an AMS^14^C age inversion was identified, either at 88 cm (16.91 kyr BP) or at 103 cm (15.8 kyr BP). Therefore, the chronology of this core was improved using the Bacon version 2.2, which is not affected by outlying (inversion) ages [9]. It uses Bayesian statistics to reconstruct accumulation histories for sedimentary deposits, considering a Student t model to address outlying (inversed) ages [9]. The Bacon version 2.2 was run within the software PaleoDataView version 0.8.3.5 [6]. The resulting age model for the core GeoB14885-1 is shown in Fig. S1. For the Irish margin core GeoB6718-2, we used a different approach. The linear interpolation of the three AMS^14^C ages reveals an unrealistic high sedimentation rate, indicative of a possible presence of inversion age. In contrast to core GeoB14885-1, core GeoB6718-2 has only three AMS^14^C ages, which hampered the usage of the Bacon software as described before. Therefore, we tuned the new AMS^14^C dates as well as the XRF (Ca(Ca+Fe)) data from core GeoB6718-2 to the nearby core GeoB6719-1 over the last 30 kyr BP [10], which showed that the age at 108 cm core depth (7 kyr) as an outlier (Fig. S2). It is noteworthy that our study focused on the last 20 kyr BP.

***Benthic foraminifera stable oxygen isotopes (δ^18^O)***

For four of the six off-mound cores used for this study, δ^18^O data were previously published and detailed information about the benthic foraminifera species used for the analyses can be found in the original references listed in S2 Table. For this study, we performed new δ^18^O analyses for off-mound core GeoB9064 (Moroccan margin, Gulf of Cadiz) and off-mound core GeoB14885-1 (Mauritanian margin; **Fig. S3**). Between 3–8 specimens of benthic foraminifera *Uvigerina* spp (≥150μm) and *Planulina ariminensis* (≥250μm), respectively, were picked in 5 cm intervals and analyzed in a Thermo Fisher Scientific 253plus gas isotope ratio mass spectrometer with a Kiel IV automated carbonate preparation device at MARUM (University of Bremen, Germany). The isotopic values were calibrated to Vienna Pee Dee Belemnite (VPDB) using the National Bureau of Standards (NBS) 18 and 19. The long-term internal δ^18^O analytical precision was ±0.06‰.

***Elemental ratios (Mg/Ca and Mn/Ca)***

All off-mound cores were analyzed for the trace metal composition of the shells of benthic foraminifera. Approximately 4–20 shells (see S5 Table for the species used) were selected every 5-cm interval and gently crushed between two glass plates to open all chambers. The samples were cleaned in three steps following the procedure of Barker et al. [11]. (1) The samples were washed five times with ultra-high-quality water (UHQ) with frequent ultrasonification after each washing and then rinsed with ethanol followed by additional ultrasonifications (two times) to remove clay mineral contamination. (2) The samples were treated with hydrogen peroxide (alkaline peroxide – H_2_O_2_ + NaOH 0,1M) in a boiling water bath for 10 minutes (every 2.5 minutes, ultrasonical cleaning for 30 seconds was applied) to eliminate organic matter. (3) A short (30s) diluted acid leaching process was applied using 0.001 M nitric acid to eliminate any adsorbed (Fe-Mn overgrowth/coating) contamination from shell fragments. Prior to the measurements, the samples were dissolved in 250µl of 0.075 M nitric acid and centrifuged. They were analyzed in replicate using an Agilent Technologies 700 Series ICP-OES Optical Emission Spectrometer provided with a Cetac ASX-520 autosampler and a micronebuliser at the MARUM Inorganic Geochemistry Laboratory (University of Bremen, Germany). Instrumental precision of the ICP-OES was monitored by analysis of an in-house standard solution with Mg/Ca ratios of 2.956 mmol/mol and Mn/Ca ratios of 0.329 mmol/mol after every five samples (long-term standard deviation of 0.003 and 0.001 mmol/mol (1sigma) respectively). To allow interlaboratory comparison we analyzed an international limestone standard (ECRM752–1) with reported Mg/Ca ratios of 3.75 mmol/mol and Mn/Ca ratios of 0.141 mmol/mol [12]. The long-term average of the ECRM752–1 standard, which is routinely analyzed twice before each batch of 50 samples in every session, is 3.762 mmol/mol for Mg/Ca ratios and 0.14 mmol/mol for Mn/Ca ratios. To avoid contamination by clay coatings and to testify the efficiency of the cleaning process, all measurements with anomalous higher Mg/Ca ratios (*i.e.* 20 mmol/mol) and with higher contents of Fe (≥1.0 mmol/mol) and Al (≥1.5 mmol/mol) were excluded in accordance with Barker et al. [11]. The low correlation of Mg/Ca ratios with Fe/Ca (R^2^ ≤0.5) and Al/Ca (R^2^ ≤0.3) ratios indicates that our Mg/Ca ratio (and, thus also Mn/Ca ratio, see below) data are not affected by any clay contaminants (**Fig. S4**).

***Bottom-water temperature and salinity reconstructions based on Mg/Ca ratios and stable oxygen isotopes***

Bottom-water temperature and salinity were reconstructed using paired measurements of the elemental Mg/Ca ratio and stable oxygen isotopes (δ^18^O) on benthic foraminifera shells (S5 Table). To account for regional environmental differences and occurrences of benthic foraminifera species as well as the lack of species-specific calibrations at some locations in the North Atlantic and, specially, in the Mediterranean Sea, the best fitting equations to estimate bottom-water temperature and salinity have been selected from the literature. The Mg/Ca values were converted into bottom-water temperature using different calibrations listed in S5 Table. Benthic foraminifera Mg/Ca-temperature calibrations are still scarce and very limited in number, species and temperature range [13–16], making the absolute bottom-water temperature reconstruction for regions without calibration (*i.e.* Irish margin/Porcupine Seabight, Moroccan margin/Gulf of Cadiz and Mediterranean Sea) difficult. To compensate for the lack of species-specific benthic foraminifera Mg/Ca-temperature calibration for *Cibicides* spp. from the Mediterranean Sea, for cores GeoB18131-1 (West Melilla) and GeoB13731-1 (East Melilla) bottom-water temperatures were reconstructed applying the paleotemperature equation from Cacho et al. [17], which modified the pervious *Cibicides* spp. calibration from Lear et al. [18]. To reconstruct the bottom-water temperatures from the Moroccan margin (Gulf of Cadiz; core GeoB9064-1), we used the *Uvigerina* spp. calibration from surface sediments of the Florida Straits [13], while for bottom-water temperature reconstructions for the Irish margin (Porcupine Seabight; core GeoB6718-2) the *Planulina ariminensis* calibration developed for the tropical NW Africa [15] was used. Absolute temperature values are dependent on the applied equation. Independently from the paleotemperature equations, benthic foraminifera Mg/Ca ratio increases as the temperature increases with a sensitivity ranging between 0.08 and 0.18 mmol/mol per ^°^C when applying the paleotemperature equations listed in S5 Table. The estimated bottom-water temperature is shown in figure S5.

The seawater δ^18^O (δ^18^O_SW_), a conventional proxy for paleosalinity, was estimated by applying the benthic foraminifera temperature-δ^18^O relationship. For the Gulf of Mexico, we applied the *Cibicidoides* spp+ *P. ariminensis* linear equation (δ^18^O_SW_ = 0.225T – 3.5 + δ^18^O_C_ +0.27) from [19]. However, for the Moroccan margin (Gulf of Cadiz), the Mauritanian margin, and the Mediterranean Sea, no calibrations are available. Therefore, we used the [20] equation as well as its modification provided by Huang et al. [15]. A summary with all equations applied at the different case study sites is given in S5 Table. The effect of ice volume-related sea level changes on δ^18^O_SW_ was removed using the sea level curve from Waelbroeck et al. [21]. The average global change in δ^18^O_SW_ since the LGM is assumed to be 1.0±0.1‰ [22]. A constant of 0.27‰ was added to convert resulting δ^18^O_SW_ values from Vienna Peedee belemnite (VPDB) into the Standard Mean Ocean Water (SMOW). The propagation uncertainty of δ^18^O_SW_ was calculated following [23].

***Bottom-water oxygenation reconstruction based on Mn/Ca ratios***

Benthic foraminifera Mn/Ca ratios have been successfully providing estimates of the bottom-water oxygen content [24–26]. Mn is a redox-sensitive element that is thermodynamically unstable is oxidized seawater, so that Mn^2+^ is oxidized and precipitated as Mn oxyhydroxides [27]. However, under low oxygen contents, it is reduced and Mn^2+^ concentration in the surrounding water increases. Thus, when living benthic foraminifera calcify their shells under poorly oxygenated waters, more Mn is incorporated in the shells and, consequently, the Mn/Ca ratio can be used as a proxy for bottom-water oxygenation [24,26]. Species-specific and regional calibrations are indispensable to reconstruct absolute oxygen concentrations, however, due to the lack of calibrations for our study locations, the Mn/Ca ratios were only used as qualitative indicators for changes in the oxygenation state of the bottom waters. It is noteworthy that benthic foraminifera Mn/Ca ratios can be also influenced by the contamination of the analyzed samples with terrigenous material [24,26]. However, as already shown in Fig. S4 for the Mg/Ca ratios, also our Mn/Ca ratios are not affected by any contamination of clay minerals as indicated by the low correlation between terrigenous elements (e.g., Al) and Mn (Al/Mn; R^2^ ≤0.01) (Fig. S6). Thus, our benthic foraminifera Mn/Ca ratios serve as good indicators for bottom-water redox conditions.

***Reconstructions of the bottom-water hydrodynamic regime based on grain-size analyses***

The grain-size distribution of the terrigenous sediment fraction is controlled by the hydrodynamic conditions at the seabed, where coarser grains indicate stronger bottom currents, while finer grains indicate weaker bottom currents [10,28]. The grain-size data for the off-mound cores used here and the details of the applied methodology were published previously (S2 Table), except for the core GeoB14885-1 (Mauritanian margin). For this core the grain-size distribution was analyzed for this study applying a sampling interval of 5 cm. Analyzes were run in the Particle-Size Laboratory at MARUM, University of Bremen, with a Beckman Coulter Laser Diffraction Particle Size Analyzer LS 13320. Prior to the measurements, the terrigenous sediment fraction was isolated by removing organic carbon, calcium carbonate, and biogenic opal by boiling the samples (in about 200 ml water) with 10 ml of H_2_O_2_ (35%; until the reaction stopped), 10 ml of HCl (10%; 1 min) and 6 g NaOH pellets (10 min), respectively. After every preparation step, the samples were diluted (dilution factor: >25). Finally, remaining aggregates were destroyed prior to the measurements by boiling the samples with ~0.3 g tetra-sodium diphosphate decahydrate (Na_4_P_2_O_7_ * 10H_2_O, 3 min) (see also McGregor et al. [29]). Sample preparation and measurements were carried out with deionized, degassed and filtered water (filter mesh size: 0.2 µm) to reduce the potential influence of gas bubbles or particles within the water. The obtained results provide the grain-size distribution of a sample from 0.04 to 2000 μm divided in 116 size classes. The calculation of the particle sizes relies on the Fraunhofer diffraction theory and the Polarization Intensity Differential Scattering (PIDS) for particles from 0.4 to 2000 µm and from 0.04 to 0.4 µm, respectively. The reproducibility is checked regularly by replicate analyses of three internal glass-bead standards and is found to be better than ±0.7 µm for the mean and ±0.6 µm for the median particle size (1σ). The average standard deviation integrated overall size classes is better than ±4 vol% (note that the standard deviation of the individual size classes is not distributed uniformly). All provided statistic values are based on a geometric statistic.

***Reconstructions of export productivity based on benthic foraminifera accumulating rates (BFAR)***

As heterotrophic organism, CWC are very sensitive to food availability by the vertical flux of phytodetritus derived from increased surface productivity and processes which deliver and/or enrich food particles [30–33]. The organic matter export flux to the seafloor was inferred based on the benthic foraminifera accumulation rate (BFAR) expressed as the number of shells cm^-2^ kyr^-1^ [34]. High accumulation of benthic foraminifera has been found in regions of high biological surface ocean production, such as upwelling zones [35]. In this context, the BFAR is positively related to organic matter fluxes to the sea floor, mainly reflecting export productivity [34,35]. Considering the high sensitivity of benthic foraminifera also to bottom water oxygenation, the lack of a correlation between BFAR and bottom-water oxygenation (Mn/Ca records) as obtained for our study locations (Fig. S7–S12) indicates that BFAR reflects the flux of organic matter to the seafloor. Therefore, it supports the BFAR as a reliable proxy to qualitatively assess changes in organic matter supply to the seafloor.

Supplementary Results

Here, we provide an additional overview of all data used ordered by the individual sediment cores.

The Gulf of Mexico (Campeche province), Northwest Atlantic

The Ireland margin (Porcupine Seabight), Northeast Atlantic

The Moroccan margin (Gulf of Cádiz), Northeastern Atlantic Ocean

The Mauritanian Margin, Eastern Tropical Atlantic Ocean

The Alboran Sea (West Melilla coral mound province), Western Mediterranean Sea

The Alboran Sea (East Melilla coral mound province), Western Mediterranean Sea

**References:**

1. Hebbeln D, Portilho-Ramos RC, Wienberg C, Titschack J. The fate of cold-water corals in a changing world: a geological perspective. Front Mar Sci. 2019;6. doi:10.3389/fmars.2019.00119

2. Garcia HE, Weathers, KW, Paver, CR, Smolyar, IV, Boyer, TP, Locarnini, RA, Zweng, MM, Mishonov, AV, Baranova, OK, Reagan, JR (2019a). World Ocean Atlas 2018, Volume 3: dissolved oxygen, apparent oxygen utilization, and dissolved oxygen saturation. A. Mishonov Technical Editor. NOAA Atlas NESDIS 83, 38pp.

3. Garcia HE, Boyer, TP, Baranova, OK, Locarnini, RA, Mishonov, AV, Grodsky, A, Paver, CR, Weathers, KW Smolyar, IV, Reagan, JR, Seidov, D, Zweng, MM (2019b). World Ocean Atlas 2018: Product Documentation. A. Mishonov, Technical Editor.

4. Locarnini, RA, Mishonov, AV, Baranova, OK, Boyer, TP, Zweng, MM, Garcia, HE, Reagan, JR, Seidov, D, Weathers, KW, Paver, CR, Smolyar, IV (2019). World Ocean Atlas 2018, Volume 1: Temperature. A. Mishonov, Technical Editor. NOAA Atlas NESDIS 81, 52pp.

5. Zweng, MM, Reagan, JR, Seidov, D, Boyer, TP, Locarnini, RA, Garcia, HE, Mishonov, AV, Baranova, OK, Weathers, KW, Paver, CR, Smolyar, IV (2019). World Ocean Atlas 2018, Volume 2: Salinity. A. Mishonov Technical Editor, NOAA Atlas NESDIS 82, 50pp.

6. Langner M, Mulitza S. Technical note: PaleoDataView – a software toolbox for the collection, homogenization and visualization of marine proxy data. Clim Past. 2019;15: 2067–2072. doi:10.5194/cp-15-2067-2019

7. Reimer PJ, Austin WEN, Bard E, Bayliss A, Blackwell PG, Bronk Ramsey C, et al. The IntCal20 northern hemisphere radiocarbon age calibration curve (0–55 cal kBP). Radiocarbon. 2020;62: 725–757. doi:10.1017/RDC.2020.41

8. Butzin M, Köhler P, Lohmann G. Marine radiocarbon reservoir age simulations for the past 50,000 years. Geophys Res Lett. 2017;44: 8473–8480. doi:10.1002/2017GL074688

9. Blaauw M, Christeny JA. Flexible paleoclimate age-depth models using an autoregressive gamma process. Bayesian Anal. 2011;6: 457–474. doi:10.1214/11-BA618

10. Dorschel B, Hebbeln D, Rüggeberg A, Dullo W, Freiwald A. Growth and erosion of a cold-water coral covered carbonate mound in the Northeast Atlantic during the Late Pleistocene and Holocene. Earth Planet Sci Lett. 2005;233: 33–44. doi:10.1016/j.epsl.2005.01.035

11. Barker S, Greaves M, Elderfield H. A study of cleaning procedures used for foraminiferal Mg/Ca paleothermometry. Geochemistry, Geophys Geosystems. 2003;4:. doi:10.1029/2003GC000559

12. Greaves M, Caillon N, Rebaubier H, Bartoli G, Bohaty S, Cacho I, et al. Interlaboratory comparison study of calibration standards for foraminiferal Mg/Ca thermometry. Geochemistry, Geophys Geosystems. 2008;9 doi:10.1029/2008GC001974

13. Bryan SP, Marchitto TM. Mg/Ca-temperature proxy in benthic foraminifera: New calibrations from the Florida Straits and a hypothesis regarding Mg/Li. Paleoceanography. 2008;23. doi:10.1029/2007PA001553

14. Elderfield H, Yu J, Anand P, Kiefer T, Nyland B. Calibrations for benthic foraminiferal Mg/Ca paleothermometry and the carbonate ion hypothesis. Earth Planet Sci Lett. 2006;250: 633–649. doi:10.1016/j.epsl.2006.07.041

15. Huang E, Mulitza S, Paul A, Groeneveld J, Steinke S, Schulz M. Response of eastern tropical Atlantic central waters to Atlantic meridional overturning circulation changes during the Last Glacial Maximum and Heinrich Stadial 1. Paleoceanography. 2012;27. doi:10.1029/2012PA002294

16. Yu J, Elderfield H. Mg/Ca in the benthic foraminifera Cibicidoides wuellerstorfi and Cibicidoides mundulus: Temperature versus carbonate ion saturation. Earth Planet Sci Lett. 2008;276: 129–139. doi:10.1016/j.epsl.2008.09.015

17. Cacho I, Shackleton N, Elderfield H, Sierro FJ, Grimalt JO. Glacial rapid variability in deep-water temperature and δ^18^O from the Western Mediterranean Sea. Quat Sci Rev. 2006;25: 3294–3311. doi:10.1016/j.quascirev.2006.10.004

18. Lear CH, Rosenthal Y, Slowey N. Benthic foraminiferal Mg/Ca-paleothermometry: a revised core-top calibration. Geochim Cosmochim Acta. 2002;66: 3375–3387. doi:10.1016/S0016-7037(02)00941-9

19. Marchitto TM, Curry WB, Lynch-Stieglitz J, Bryan SP, Cobb KM, Lund DC. Improved oxygen isotope temperature calibrations for cosmopolitan benthic foraminifera. Geochim Cosmochim Acta. 2014;130: 1–11. doi:10.1016/j.gca.2013.12.034

20. Schakleton NJ. Attainment of isotopic equilibrium between ocean water and the benthonic foraminifera genus Uvigerina: isotopic changes in the ocean during the last glacial. 1974.

21. Waelbroeck C, Labeyrie L, Michel E, Duplessy JC, McManus JF, Lambeck K, et al. Sea-level and deep water temperature changes derived from benthic foraminifera isotopic records. Quat Sci Rev. 2002;21: 295–305. doi:10.1016/S0277-3791(01)00101-9

22. Schrag DP, Adkins JF, McIntyre K, Alexander JL, Hodell DA, Charles CD, et al. The oxygen isotopic composition of seawater during the Last Glacial Maximum. Quat Sci Rev. 2002;21: 331–342. doi:10.1016/S0277-3791(01)00110-X

23. Mohtadi M, Prange M, Oppo DW, De Pol-Holz R, Merkel U, Zhang X, et al. North Atlantic forcing of tropical Indian Ocean climate. Nature. 2014;509: 76–80. doi:10.1038/nature13196

24. Glock N, Eisenhauer A, Liebetrau V, Wiedenbeck M, Hensen C, Nehrke G. EMP and SIMS studies on Mn/Ca and Fe/Ca systematics in benthic foraminifera from the Peruvian OMZ: a contribution to the identification of potential redox proxies and the impact of cleaning protocols. Biogeosciences. 2012;9: 341–359. doi:10.5194/bg-9-341-2012

25. Groeneveld J, Filipsson HL. Mg/Ca and Mn/Ca ratios in benthic foraminifera: the potential to reconstruct past variations in temperature and hypoxia in shelf regions. Biogeosciences. 2013;10: 5125–5138. doi:10.5194/bg-10-5125-2013

26. Groeneveld J, Filipsson HL, Austin WEN, Darling K, McCarthy D, Quintana Krupinski NB, et al. Assessing proxy signatures of temperature, salinity, and hypoxia in the Baltic Sea through foraminifera-based geochemistry and faunal assemblages. J Micropalaeontology. 2018;37: 403–429. doi:10.5194/jm-37-403-2018

27. Tribovillard N, Algeo TJ, Lyons T, Riboulleau A. Trace metals as paleoredox and paleoproductivity proxies: Chem Geol. 2006;232: 12–32. doi:10.1016/j.chemgeo.2006.02.012

28. McCave IN, Thornalley DJR, Hall IR. Relation of sortable silt grain-size to deep-sea current speeds: Calibration of the ‘Mud Current Meter’. Deep Sea Res Part I Oceanogr Res Pap. 2017;127: 1–12. doi:10.1016/j.dsr.2017.07.003

29. McGregor H V., Dupont L, Stuut J-BW, Kuhlmann H. Vegetation change, goats, and religion: a 2000-year history of land use in southern Morocco. Quat Sci Rev. 2009;28: 1434–1448. doi:10.1016/j.quascirev.2009.02.012

30. Davies AJ, Guinotte JM. Global Habitat Suitability for Framework-Forming Cold-Water Corals. PLoS One. 2011;6: e18483. doi:10.1371/journal.pone.0018483

31. Hebbeln D, Van Rooij D, Wienberg C. Good neighbours shaped by vigorous currents: Cold-water coral mounds and contourites in the North Atlantic. Mar Geol. 2016;378: 171–185. doi:10.1016/j.margeo.2016.01.014

32. Mienis F, de Stigter HC, White M, Duineveld G, de Haas H, van Weering TCE. Hydrodynamic controls on cold-water coral growth and carbonate-mound development at the SW and SE Rockall Trough Margin, NE Atlantic Ocean. Deep Sea Res Part I Oceanogr Res Pap. 2007;54: 1655–1674. doi:10.1016/j.dsr.2007.05.013

33. Thiem Ø, Ravagnan E, Fosså JH, Berntsen J. Food supply mechanisms for cold-water corals along a continental shelf edge. J Mar Syst. 2006;60: 207–219. doi:10.1016/j.jmarsys.2005.12.004

34. Herguera JC, Berger WH. Paleoproductivity from benthic foraminifera abundance: Glacial to postglacial change in the west-equatorial Pacific. Geology. 1991;19: 1173. doi:10.1130/0091-7613(1991)019<1173:PFBFAG>2.3.CO;2

35. Eberwein A, Mackensen A. Regional primary productivity differences off Morocco (NW-Africa) recorded by modern benthic foraminifera and their stable carbon isotopic composition. Deep Res Part I Oceanogr Res Pap. 2006;53: 1379–1405. doi:10.1016/j.dsr.2006.04.001

36. Matos L, Wienberg C, Titschack J, Schmiedl G, Frank N, Abrantes F, et al. Coral mound development at the Campeche cold-water coral province, southern Gulf of Mexico: Implications of Antarctic Intermediate Water increased influence during interglacials. Mar Geol. 2017;392: 53–65. doi:10.1016/j.margeo.2017.08.012

37. Wienberg C, Frank N, Mertens KN, Stuut J-B, Marchant M, Fietzke J, et al. Glacial cold-water coral growth in the Gulf of Cádiz: Implications of increased palaeo-productivity. Earth Planet Sci Lett. 2010;298: 405–416. doi:10.1016/j.epsl.2010.08.017

38. Wienberg C, Hebbeln D, Fink HG, Mienis F, Dorschel B, Vertino A, et al. Scleractinian cold-water corals in the Gulf of Cádiz—First clues about their spatial and temporal distribution. Deep Sea Res Part I Oceanogr Res Pap. 2009;56: 1873–1893. doi:10.1016/j.dsr.2009.05.016

39. Wang H, Lo Iacono C, Wienberg C, Titschack J, Hebbeln D. Cold-water coral mounds in the southern Alboran Sea (western Mediterranean Sea): Internal waves as an important driver for mound formation since the last deglaciation. Mar Geol. 2019;412: 1–18. doi:10.1016/j.margeo.2019.02.007

40. Fink HG, Wienberg C, De Pol-Holz R, Wintersteller P, Hebbeln D. Cold-water coral growth in the Alboran Sea related to high productivity during the Late Pleistocene and Holocene. Mar Geol. 2013;339: 71–82. doi:10.1016/j.margeo.2013.04.009

41. Stuiver M, Reimer PJ, Reimer RW (2021). CALIB 8.2 at http://calib.org, accessed 2021-10-29

42. Heaton TJ, Köhler P, Butzin M, Bard E, Reimer RW, Austin WEN, et al. Marine20—the marine radiocarbon age calibration curve (0–55,000 CAL BP). 2020;62: 779–820. doi:10.1017/RDC.2020.68

43. Frank N, Paterne M, Ayliffe L, van Weering T, Henriet JP, Blamart D. Eastern North Atlantic deep-sea corals: tracing upper intermediate water Δ^14^C during the Holocene. Earth Planet Sci Lett. 2004;219: 297–309. doi:10.1016/S0012-821X(03)00721-0

44. Frank N, Lutringer A, Paterne M, Blamart D, Henriet J-P, van Rooij D, et al. Deep-water corals of the northeastern Atlantic margin: carbonate mound evolution and upper intermediate water ventilation during the Holocene. Cold-water corals and Ecosystems. Berlin, Heidelberg: Springer Berlin Heidelberg; 2005. pp. 113–133. doi:10.1007/3-540-27673-4_6

45. Muller-Karger FE, Varela R, Thunell R, Luerssen R, Hu C, Walsh JJ. The importance of continental margins in the global carbon cycle. Geophys Res Lett. 2005;32: 1–4. doi:10.1029/2004GL021346

46. Reimer PJ, McCormac FG. Marine radiocarbon reservoir corrections for the Mediterranean and Aegean Seas. Radiocarbon. 2002;44: 159–166. doi:10.1017/S0033822200064766

47. Siani G, Paterne M, Michel E, Sulpizio R, Sbrana A, Arnold M, et al. Mediterranean sea surface radiocarbon reservoir age changes since the last glacial maximum. Science (80- ). 2001;294: 1917–1920. doi:10.1126/science.1063649

48. Siani G, Paterne M, Arnold M, Bard E, Métivier B, Tisnerat N, et al. Radiocarbon reservoir ages in the Mediterranean Sea and Black Sea. Radiocarbon. 2000;42: 271–280. doi:10.1017/S0033822200059075

49. Wienberg C, Titschack J, Frank N, De Pol-Holz R, Fietzke J, Eisele M, et al. Deglacial upslope shift of NE Atlantic intermediate waters controlled slope erosion and cold-water coral mound formation (Porcupine Seabight, Irish margin). Quat Sci Rev. 2020;237: 106310. doi:10.1016/j.quascirev.2020.106310

50. Frank N, Ricard E, Lutringer-Paquet A, van der Land C, Colin C, Blamart D, et al. The Holocene occurrence of cold water corals in the NE Atlantic: Implications for coral carbonate mound evolution. Mar Geol. 2009;266: 129–142. doi:10.1016/j.margeo.2009.08.007

51. Schröder-Ritzrau A, Freiwald A, Mangini A. U/Th-dating of deep-water corals from the eastern North Atlantic and the western Mediterranean Sea. Cold-water corals and Ecosystems. Berlin/Heidelberg: Springer-Verlag; 2005. pp. 157–172. doi:10.1007/3-540-27673-4_8

52. Raddatz J, Rüggeberg A, Liebetrau V, Foubert A, Hathorne EC, Fietzke J, et al. Environmental boundary conditions of cold-water coral mound growth over the last 3 million years in the Porcupine Seabight, Northeast Atlantic. Deep Sea Res Part II Top Stud Oceanogr. 2014;99: 227–236. doi:10.1016/j.dsr2.2013.06.009

53. Eisele M, Hebbeln D, Wienberg C. Growth history of a cold-water coral covered carbonate mound — Galway Mound, Porcupine Seabight, NE-Atlantic. Mar Geol. 2008;253: 160–169. doi:10.1016/j.margeo.2008.05.006

54. Frank N, Freiwald A, López Correa M, Wienberg C, Eisele M, Hebbeln D, et al. Northeastern Atlantic cold-water coral reefs and climate. Geology. 2011;39: 743–746. doi:10.1130/G31825.1

55. Wienberg C, Titschack J, Freiwald A, Frank N, Lundälv T, Taviani M, et al. The giant Mauritanian cold-water coral mound province: Oxygen control on coral mound formation. Quat Sci Rev. 2018;185: 135–152. doi:10.1016/j.quascirev.2018.02.012

56. Eisele M, Frank N, Wienberg C, Hebbeln D, López Correa M, Douville E, et al. Productivity controlled cold-water coral growth periods during the last glacial off Mauritania. Mar Geol. 2011;280: 143–149. doi:10.1016/j.margeo.2010.12.007

57. Stalder C, Vertino A, Rosso A, Rüggeberg A, Pirkenseer C, Spangenberg JE, et al. Microfossils, a key to unravel cold-water carbonate mound evolution through time: evidence from the Eastern Alboran Sea. Abramovich S, editor. PLoS One. 2015;10: e0140223. doi:10.1371/journal.pone.0140223

58. Fentimen R, Feenstra E, Rüggeberg A, Vennemann T, Hajdas I, Adatte T, et al. Cold-water coral mound archive provides unique insights into intermediate water mass dynamics in the Alboran Sea during the last deglaciation. Front Mar Sci. 2020;7: 1–25. doi:10.3389/fmars.2020.00354

59. Dubois-Dauphin Q, Montagna P, Siani G, Douville E, Wienberg C, Hebbeln D, et al. Hydrological variations of the intermediate water masses of the western Mediterranean Sea during the past 20 ka inferred from neodymium isotopic composition in foraminifera and cold-water corals. Clim Past. 2017;13: 17–37. doi:10.5194/cp-13-17-2017

60. Shackleton NJ. Attainment of isotopic equilibrium between ocean water and the benthonic foraminifera geuns *Uvigerina*: Isotopic changes in the ocean during the last glacial. Colloq Int du CNRS. 1974;219: 203–210.
